# ASK1-K716R reduces neuroinflammation and white matter injury via preserving blood–brain barrier integrity after traumatic brain injury

**DOI:** 10.1186/s12974-023-02923-6

**Published:** 2023-10-24

**Authors:** Shan Meng, Hui Cao, Yichen Huang, Ziyu Shi, Jiaying Li, Yana Wang, Yue Zhang, Shuning Chen, Hong Shi, Yanqin Gao

**Affiliations:** 1 State Key Laboratory of Medical Neurobiology, MOE Frontiers Center for Brain Science, and Institutes of Brain Science, Fudan University, Shanghai, 200032 China; 2 Department of Anesthesiology, Shanghai Pulmonary Hospital, School of Medicine, Tongji University, Shanghai, 200433 China

**Keywords:** ASK1, JNK/p38, Blood–brain barrier integrity, Cerebral microvessel, Neurobehaviors

## Abstract

**Background:**

Traumatic brain injury (TBI) is a significant worldwide public health concern that necessitates attention. Apoptosis signal-regulating kinase 1 (ASK1), a key player in various central nervous system (CNS) diseases, has garnered interest for its potential neuroprotective effects against ischemic stroke and epilepsy when deleted. Nonetheless, the specific impact of ASK1 on TBI and its underlying mechanisms remain elusive. Notably, mutation of ATP-binding sites, such as lysine residues, can lead to catalytic inactivation of ASK1. To address these knowledge gaps, we generated transgenic mice harboring a site-specific mutant ASK1 Map3k5-e (K716R), enabling us to assess its effects and elucidate potential underlying mechanisms following TBI.

**Methods:**

We employed the CRIPR/Cas9 system to generate a transgenic mouse model carrying the ASK1-K716R mutation, aming to investigate the functional implications of this specific mutant. The controlled cortical impact method was utilized to induce TBI. Expression and distribution of ASK1 were detected through Western blotting and immunofluorescence staining, respectively. The ASK1 kinase activity after TBI was detected by a specific ASK1 kinase activity kit. Cerebral microvessels were isolated by gradient centrifugation using dextran. Immunofluorescence staining was performed to evaluate blood–brain barrier (BBB) damage. BBB ultrastructure was visualized using transmission electron microscopy, while the expression levels of endothelial tight junction proteins and ASK1 signaling pathway proteins was detected by Western blotting. To investigate TBI-induced neuroinflammation, we conducted immunofluorescence staining, quantitative real-time polymerase chain reaction (qRT-PCR) and flow cytometry analyses. Additionally, immunofluorescence staining and electrophysiological compound action potentials were conducted to evaluate gray and white matter injury. Finally, sensorimotor function and cognitive function were assessed by a battery of behavioral tests.

**Results:**

The activity of ASK1-K716R was significantly decreased following TBI. Western blotting confirmed that ASK1-K716R effectively inhibited the phosphorylation of ASK1, JNKs, and p38 in response to TBI. Additionally, ASK1-K716R demonstrated a protective function in maintaining BBB integrity by suppressing ASK1/JNKs activity in endothelial cells, thereby reducing the degradation of tight junction proteins following TBI. Besides, ASK1-K716R effectively suppressed the infiltration of peripheral immune cells into the brain parenchyma, decreased the number of proinflammatory-like microglia/macrophages, increased the number of anti-inflammatory-like microglia/macrophages, and downregulated expression of several proinflammatory factors. Furthermore, ASK1-K716R attenuated white matter injury and improved the nerve conduction function of both myelinated and unmyelinated fibers after TBI. Finally, our findings demonstrated that ASK1-K716R exhibited favorable long-term functional and histological outcomes in the aftermath of TBI.

**Conclusion:**

ASK1-K716R preserves BBB integrity by inhibiting ASK1/JNKs pathway in endothelial cells, consequently reducing the degradation of tight junction proteins. Additionally, it alleviates early neuroinflammation by inhibiting the infiltration of peripheral immune cells into the brain parenchyma and modulating the polarization of microglia/macrophages. These beneficial effects of ASK1-K716R subsequently result in a reduction in white matter injury and promote the long-term recovery of neurological function following TBI.

**Supplementary Information:**

The online version contains supplementary material available at 10.1186/s12974-023-02923-6.

## Highlights

1. This study highlights the critical role of ASK1-K716’s site in regulating ASK1 activity during TBI.
2. ASK1-K716R enhances BBB integrity by inhibiting ASK1/JNKs activity in endothelial cells, thereby reducing the degradation of tight junction proteins following TBI.
3. ASK1-K716R suppresses the infiltration of peripheral immune cells and attenuates microglia-mediated neuroinflammation following TBI.
4. ASK1-K716R mitigates white matter injury and facilitates the long-term recovery of neurological function following TBI.

**Supplementary Information:**

The online version contains supplementary material available at 10.1186/s12974-023-02923-6.

## Background

Traumatic brain injury (TBI) represents as a significant public health challenge worldwide, contributing to a high burden of morbidity and mortality. Each year, over 27 million new TBI cases are diagnosed globally, with more than one million occuring in the United States alone [1]. TBI is regarded as an acute insult to the brain, leading to a cascade of pathological events that can result in long-term chronic sequelae and neurodegeneration [2]. Despite recent advancements in both experimental and clinical neuroscience, effective strategies for preventing comprehensive secondary injuries following TBI remain limited, underscoring the need for novel therapeutic interventions.

Blood–brain barrier (BBB) disruption and the accompanying inflammatory responses are immediate consequences triggered by TBI that can persist over extended periods [3, 4]. The breakdown of the BBB leads to the accumulation of harmful metabolites and facilitates the infiltration of peripheral immune cells into the injured brain regions [5–7]. The release of inflammatory cytokines by peripheral immune cells and damage-associated molecular patterns (DAMPs) from damaged brain cells exacerbates resident microglia-mediated neuroinflammation [8–11]. The deteriorating microenvironment caused by BBB disruption and neuroinflammation perpetuates a cycle that accelerates neuronal death and white matter injury, creating a detrimental feedback loop. Importantly, the effects of BBB breakdown and neuroinflammation have enduring consequences and are significant therapeutic targets in the management of TBI due to their association with neurodegeneration and other comorbidities [12].

Apoptosis signal-regulated kinase 1 (ASK1), a mitogen-activated protein kinase (MAPK) kinase kinase, plays a crucial role in various cell physiological and pathological processes by activating the c-Jun N-terminal kinases (JNKs) and p38 MAPK signaling pathways. ASK1 signaling has been implicated in neuronal death and neuroinflammation in acute brain injuries, such as ischemic stroke [13]. Additionally, studies have demonstrated that the knockout of ASK1 in microglia attenuates neurological impairment and inflammatory responses in a murine model of epileptic seizures [14]. However, the potential neuroprotective role of ASK1 inhibition in TBI remains unclear.

As a MAPK kinase kinase, ASK1 functions by transferring a phosphate group of an ATP onto the substrate kinase. Notably, specific mutations at ATP-binding sites, such as lysine residues, can lead to the catalytic inactivation of ASK1 [15, 16]. In alignment with the murine ASK1 sequence available in the UniProt database (https://www.uniprot.org/uniprotkb/O35099/entry), we identified an ATP-binding site at the kinase domain (KD) located at Lys716. This site serves as a potential target for regulating the activity of ASK1. In this study, we utilized the CRIPR/Cas9 system to introduce target mutations into ASK1-KD, generating an ASK1- K716R transgenic mouse model. Through this transgenic approach, we observed a significant reduction in ASK1 kinase activity, leading to the attenuation of downstream JNKs and p38 pathways following TBI. Notably, ASK1-K716R exhibited a protective effect by reducing cerebral vascular endothelial apoptosis and preserving BBB integrity by inhibiting ASK1/JNKs pathway. Moreover, ASK1-K716R also exhibited neuroprotective properties by decreasing neuroinflammation and white matter injury, while simultaneously improving long-term functional and histological outcomes following TBI.

## Materials and methods

### Experimental animals and grouping

ASK1 Map3k5-e (K716R) site-specific mutant mice were generated through custom services provided by Shanghai Biomodel Organism Science & Technology Development Co., Ltd. The CRIPR/Cas9 system was employed to induce homologous recombination repair and introduce the target mutation. Fertilized eggs from C57BL/6J mice were microinjected with Cas9 mRNA, guide RNA, and oligo donor DNA to facilitate homologous recombination repair and introduce the target mutation. PCR analysis was performed to confirm the presence of ASK1-K716R mutation in the F0 generation mice. Heterozygous F1 generation mice were obtained by mating the F0 generation mice with C57BL/6J mice. Subsequently, homozygous ASK1 Map3k5-e site-specific mutant mice were obtained by mating the F1 generation mice. After every two backcrosses of genetically mutated mice, 2–3 mice were randomly selected for DNA sequencing to monitor off-target mutation. C57BL/6J mice, purchased from the same supplier, were used as the wild-type mice in this study.

Ethical approval for all animal experiments was obtained from the Animal Care and Use Committee of Shanghai Medical College, Fudan University (approval number 2018-JS-003). Mice aged 8–10 weeks were housed under standard conditions with a controlled temperature of 22–25 °C and a 12 h light–dark cycle, with ad libitum access to food and water. The mice were randomly assigned to four groups: wild-type sham (WT sham), ASK1-K716R sham (ASK1-K716R sham), wild-type TBI (WT TBI), ASK1-K716R TBI (ASK1-K716R TBI).

### TBI model

TBI was induced using a Controlled cortical impact (CCI) device (TBI 0310, Precision Systems and Instrumentation). Mice were anesthetized with 1–2% isoflurane (RWD, Shanghai, China) in 70% N_2_/30% O_2_ mixture and securely fixed on the stereotaxic apparatus. A midline incision was made and a 4-mm craniotomy was performed over the right parietotemporal cortex using a motorized drill. The center of CCI was positioned at 2.0 mm lateral to the midline and 0.5 mm anterior to bregma, using a 3.0 mm flat-tip impounder (velocity, 3.5 m/s; duration, 150 ms; depth, 1.5 mm). Following CCI, the scalp incision was sutured and the animals were kept on a heating pad until they regained consciousness. Sham mice underwent all these procedures, except the CCI impact, to serve as a control group.

### ASK1 kinase activity detection

Mice brains were carefully harvested under anesthetic and subsequently homogenized. ASK1 enrichment was achieved using SureBeads G Beads (#1614021, Bio-Rad) and rabbit anti-ASK1 (1:1000, 3762, Cell signaling Technology). ASK1 kinase activity was assessed using the HTRF KinEASE-STK S3 kit (62STPEB, Cisbo) according to the manufacturer’s instructions.

### Western blotting

Total protein was extracted using cold RIPA buffer (9803S, Cell signaling Technology, USA) supplemented with protease inhibitor cocktails (Roche). Equal amounts of protein were loaded onto SDS-PAGE gels and transferred onto polyvinylidene difluoride (PVDF) membranes (Merck, Germany). After blocking with 5% BSA for 1 h, the PVDF membrane was incubated overnight at 4 °C with primary antibodies and then with secondary antibodies for 1 h at room temperature the next day. Primary antibodies used included rabbit anti-pASK1 (1:300, 3765, Cell signaling Technology, USA), rabbit anti-ASK1 (1:1000, 3762, Cell signaling Technology, USA), rabbit anti-pSAPK/JNKs (1:1000,9255, Cell signaling Technology, USA), rabbit anti-SAPK/JNKs (1:1000, 9252, Cell signaling Technology, USA), rabbit anti-pp38 (1:1000,9215, Cell signaling Technology, USA), rabbit anti-p38 (1:1000, 9212, Cell signaling Technology, USA), rabbit anti-VE-cadherin (1:1000, ab205336, Abcam, USA), rabbit anti-Occludin (1:1000, ab216327, Abcam, USA), rabbit anti-MMP9 (1:1000, ab38898, Abcam, USA), mouse anti-β-actin (1:10,000, 4967, Cell signaling Technology, USA). The secondary antibodies used were HRP-conjugated goat anti-mouse IgG (1:2000, 7076, Cell signaling Technology, USA) and HRP-conjugated goat anti-rabbit IgG (1:2000, 7074, Cell signaling Technology, USA). Protein bands were visualized using the ChemiDoc MP System (Bio-Rad, USA) and quantified for intensity using ImageJ software (National Institutes of health, Bethesda, MD, USA).

### Immunofluorescence, TUNEL staining and image analysis

After anesthetized deeply, mice were transcardially perfused with ice-cold PBS followed by 4% paraformaldehyde to fix the brains. Brains were then removed and sequentially immersed in 4% paraformaldehyde, 20% sucrose, and 30% sucrose to facilitate fixation and dehydration. Coronal brain sections with a thickness of 25-μm were sliced using the freezing microtome (HM525NX, ThermoFisher, USA). After washing with PBS and PBS + 0.3% Triton, brain sections were blocked with 10% goat serum or donkey serum for 1 h. Subsequently, the brain sections were incubated overnight at 4 °C with the following primary antibodies: anti-NeuN (1:1000, ab177487, abcam), anti-MBP (1:500, ab40390, abcam), anti-Iba1 (1:1000, ab5076, abcam), anti-SMI32 (1:200, 801701, BioLegend), anti-NF200 (1:200, ab82259, abcam), anti-Iba1 (1:1000, ab5076, abcam), anti-Caspr (1:1000, MABN69, MilliporeSigma), anti-Nav1.6 (1:1000, ASC-009, Alomone Labs), anti-CD16/32 (1:1000, ab25235, abcam), anti-CD206 (1:1000, 17–7321-82, R&D), anti-CD31 (1:1000, AF3628, R&D), anti-GFAP (1:1000, ab4674, abcam), anti-pp38 (1:1000, AF4001, Affnity), anti-VE-Cadherin (1:1000, ab205336, abcam), anti-Occludin (1:1000, ab216327, abcam), anti-pJNKs (1:1000, AF3318, Affnity), anti-pASK1 (1:1000, AF3477, Affinity). After washing, the brain sections were incubated with appropriate Alexa-Fluor-conjugated secondary antibodies (1:1000; Jackson ImmunoResearch, USA) for 1 h at room temperature and cover-slipped with DAPI-Fluoromount-G (Sigma, USA).

For terminal deoxynucleotidyl transferase dUTP nick end labelling (TUNEL) staining, a TUNEL apoptosis assay kit (Beyotime, China) was used following the manufacturer’s instructions. Briefly, after washing, brain sections were permeabilized with 1% Triton X-100 for 10 min at 4 °C, and then incubated with the TUNEL reaction mixture for 60 min at 37 °C in the dark.

Sections were captured using a laser scanning confocal microscope (Nikon A1, Germany) and analyzed using Image J software (version 1.8.0, National Institutes of health, Bethesda, MD, USA). Imaris software was used to reconstruct 3-dimensional (3D) images of confocal z-stacks. Specifically, a specific region was selected to establish the surface, and with an appropriate threshold set, each channel signal was reconstructed into a 3D model. All images were processed consistently.

### Assessment of BBB impairment

BBB permeability was assessed by measuring the extravasation of exogenous Evans blue (EB), leakage of fluorescent tracer Alexa 555 cadaverine, and endogenous plasma IgG into the brain parenchyma following TBI.

Mice were anesthetized and intravenously injected with 4% EB (E2129, Sigma, 4 mg/kg) 72 h after TBI. Following a 2 h exposure to EB, mice were anesthetized, and intracardiac perfusion with PBS was conducted, followed by brain tissue collection. The ipsilateral hemisphere was weighed, and then incubated at 4 ℃ after homogenization at 50% trichloroacetic acid (TCA) for 24 h. The TCA absorbance at 620 nm was measured after centrifugation and compared with a standard curve.

For cadaverine and IgG leakage measurements, mice received a femoral vein injection of 200 μl Alexa Flour 555-cadaverin (0.95 kDa, Invitrogen, 1μg/μl) 1 h before euthanasia. After intracardiac perfusion and fixation with 4% paraformaldehyde, brains were dissected into 25 μm coronal sections using a freezing microtome (HM525NX, ThermoFisher, USA). The presence of Alexa 555 cadaverine was observed as red fluorescence in the brain tissue under the microscope. Sections were either directly processed for fluorescence detection or subjected to immunofluorescent labeling for IgG using anti-mouse IgG Cy3. The leakage volume of cadaverine was calculated by measuring its area on ten equally spaced brain sections and multiplied by 0.25 μm. Similarly, the leakage area of IgG was obtained from ten brain sections. Additionally, the fluorescence intensity of IgG at a high level was displayed using the 3D surface plot of Image J.

### Isolation of total RNA and quantitative real-time PCR (qPCR)

Total RNA isolation from tissue samples was carried out using Trizol reagent (19201ES60, Yeasen, Shanghai, China). cDNA synthesis for mRNA was performed using the First Strand cDNA Synthesis Kit (K1622, Thermo Fisher Scientific, Pittsburgh, PA, USA). Real-time qPCR was conducted on a 7500 Real-Time PCR System (Applied Biosystems; Thermo Fisher Scientific, Inc.) using HiffTM QPCR SYBR® Green Master Mix (11201ES08, Yeasen, Shanghai, China) as the detection dye. The PCR conditions involved 40 cycles of 95 ℃ for 30 s, 60 ℃ for 34 s, and 72 ℃ for 30 s. Additional file 1: All the primer sequences are in the Table S1 of the Supplementary materials file. The relative expression levels of the candidate genes were analyzed using the 2-ΔΔCt method.

### Electrophysiology

Compound action potentials (CAPs) in the external capsule were recorded [17]. Briefly, mice were sacrificed under deep anesthesia to rapidly collect brain samples. Coronal brain slices of 350-μm thickness were obtained using a vibratome (1200 s, Leica). These slices were then transferred to artificial cerebrospinal fluid (aCSF) consisting 124 mM NaCl, 2.5 mM KCl, 2 mM CaCl_2_, 1 mM NaH_2_PO_4_, 24 mM NaHCO_3_, 1.3 mM MgSO_4_, and 10 mM D-glucose. Subsequently, the slices were incubated in oxygenated aCSF with a mixture of 95% O_2_ + 5% CO_2_ at 32 °C for 0.5 h, followed by recovery at room temperature for 1 h. CAPs were induced using monophasic square waves (0.1 ms) with a concentric stimulating electrode and a glass microelectrode (5 ~ 8 MΩ). A stimulus generator (STG 4002, Multichannel) was employed for this purpose. The resulting signals were amplified using an Axoclamp 700B (Molecular Devices) and digitized using an Axon Digidata 1440A (Molecular Devices).

### Transmission electron microscopy

Fresh brain tissue (thickness < 1 mm) was fixed with 2.5% glutaraldehyde at 4 °C for 24 h. After dehydrated in graded ethanol and acetone, the brain tissue was embedded in epoxy resin and sectioned into 50-thickness slices. The prepared ultrathin sections were stained with slices, and finally stained with 3% uranium acetate and lead citrate. Subsequently, three images from each mouse were captured by Philips CM120 electron microscope and analyzed.

### Isolation of cerebral microvessels

The isolation procedure was performed as previously described [18]. In brief, the cortex of fresh brain tissue was isolated and placed in DPBS. After homogenization using a loose-fitting Dounce grinder, myelin and parenchymal cells were removed. The resulting precipitation was then suspended by 15% dextran (70 kDa) for gradient centrifugation. The pellet was transferred through a 40 μm cell strainer to collect the microvessels. These microvessels were collected in MCDB131 medium containing 0.5% BSA. The suspension was subsequently centrifuged and used for Western Blot analysis.

### Flow cytometry

Peripheral immune cell infiltration was analyzed as described previously [19]. Briefly, blood and brain were collected from Sham or TBI mice at 3 days following TBI. Mice were euthanized and peripheral blood was obtained by cardiac puncture. And then mice were perfused with cold Hank’s balanced salt solution (HBSS) to collect the impacted hemisphere. The impacted hemispheres were digested with Neural Tissue Dissociation Kit (Miltenyi) using gentleMACS Octo Dissociator with heaters (Miltenyi). The resulting homogenate was passed through a 70 μm cell strainer, and Percoll was added to create a 30% and 70% percoll gradient with distinct separation lines. After centrifugation and removal of debris, the precipitate was resuspended. Then cells were stained at 4 ℃ in darkness for 30 min with the following fluorophore-conjugated antibodies: CD11b-APC-eFlour 780 (eBioscience, 47–0112-82, 1:200), CD45-eFlour 450 (eBioscience, 48–0451-82, 1:50), Ly6G(Gr1)-PE(eBioscience, 12–9669-82, 1:200), F4/80-PE (eBioscience, 12–4801-82, 1:200), CD11c-PerCP cy5.5(eBioscience, 45-0114-82, 1:100) Ly-6G-FITC (Thermo Fisher Scientific, 11-9668-82, 1:200), CD3-APC (eBioscience, 17–0032-82, 1:200), CD19-FITC (BD Bioscience, 561740, 1:200). Flow cytometry was performed using the Beckman CytoFlex, and the obtained data were analyzed using FlowJo software.

### Measurement of tissue loss

The animals were euthanized and subsequently perfused with phosphate-buffered saline (PBS, pH 7.4) followed by 4% paraformaldehyde (w/v, dissolved in PBS). Brains were then harvested, post-fixed in 4% paraformaldehyde overnight at 4 °C, and cryoprotected in 30% sucrose in PBS for 2 days at 4 °C. Frozen serial coronal brain slices with a thickness of 25 μm were obtained using a freezing microtome (Leica). Ten equally spaced coronal brain sections ranging from bregma 1.1 to − 1.94 were subjected to immunohistochemically staining with a rabbit anti-NeuN antibody (1:1000, ab177487, Abcam, Cambridge, MA, USA). The extent of brain tissue loss was assessed on each section with Image J image analysis software (National Institutes of health, Bethesda, MD, USA), and the volume of tissue loss was calculated by numerical integration of the value. This was achieved by subtracting the volume of non-infarcted tissue in the ipsilateral hemisphere from the volume of tissue in the contralateral hemisphere. Quantification of cerebral tissue loss was conducted by unbiased Investigators who were blind to group assignments. Data were presented as the volume of tissue loss as a percentage of contralateral hemisphere volume.

### Neurobehavioral assessments

All behavioral tests were conducted by an experimenter blinded to the experimental conditions between the hours of 9:00 AM and 17:00 PM. To minimize the effects of novelty and stress, mice were placed in the testing room 30 min prior to the start of tests for habituation.

### Modified Garcia score test

Long-term neurofunctional impairment was evaluated using the modified Garcia score test, following previously established protocols [20]. The modified Garcia Neurological scores consist of several individual tests, including spontaneous activity, axial sensation, vibrissae proprioception, limb symmetry, lateral turning, forelimb walking, and climbing. Each subtest was scored on a scale of 0 to 3, and the total score was calculated by summing the scores of the seven subtests. 

### Adhesive removal test

To assess sensory and motor function impairment, mice had adhesive tapes (3 × 3 cm) applied to their ipsilateral forepaw. The latency to touch and remove the tape was measured within 120 s, with a maximum score of 120 s assigned if the tape was not removed. Three trials were conducted for each mouse, and the data were expressed as the average value of the three trials. Mice were pre-trained 1–3 d before TBI, and the data collected 1 d prior to TBI were considered as the preoperative data (pre). Repeated testing was performed at 3 d, 5 d, 7 d, 14 d, 21 d, 28 d, and 35 d after CCI.

### Grid walking test

A stainless steel frame consisting of 4 × 4 cm square grids, measuring 40 cm in length, 20 cm in width, and positioned 30 cm above the ground, was used for test. The mouse was gently placed on the frame and allowed to walk freely. Mice were pre-trained 1–3 d before TBI, and the data collected 1 d before TBI were considered as the baseline data (pre). Repeated testing was conducted at 3 d, 5 d, 7 d, 14 d, 21 d, 28 d, and 35 d after TBI. The movement of each mouse was recorded by a camera. A foot-fault step was defined as a limb falling through the grid while walking. The foot fault rate for each specific limb was calculated using the following formula: foot-fault steps / total number of steps by the limb × 100%, during a 3-min videotaped observation period.

### Rotarod test

The test was performed using the 47,650 Mouse Rota-Rod (Ugo Basile Srl, Varese, Italy). After placing the mice on the rods, the rotation speed gradually increased from 5 to 40 rotations per minute within 300 s. The latency to fall off the rod was recorded in 3 trials with an interval of 15 min. Data were expressed as the average value from 3 trials. Mice were pre-trained 1–3 d before TBI, and the data collected 1 d before TBI were considered as baseline data (pre). Repeated testing was conducted 3 d, 5 d, 7 d, 14 d, 21 d, 28 d, and 35 d after TBI.

### Wire-hanging test

The test apparatus consisted of a 50 cm long, 2 mm in diameter steel wire positioned 40 cm above the ground, with two platforms at each end. The animals were placed on the middle of the wire using their forepaws and were observed for 1 min with an interval of 5 min between three trials. The mice were scored based on the following criteria: 0, fell off; 1, hung onto the wire with both forepaws; 2, hung onto the wire with attempts to climb onto the wire; 3, hung onto the wire with both forepaws and 1 or 2 hind paws; 4, hung onto the wire with all 4 paws and the tail wrapped around the wire; and 5, escaped to one of the platforms. Repeated testing was conducted 3–35 d after TBI.

### Three‑chamber sociability and social novelty test (TCSSNT)

The test was conducted in a rectangular box (40 cm × 60 cm × 40 cm) divided into three equal boxes. Empty cages were placed in the left and right boxes to house unfamiliar mice. During the habituation period, a test mouse was placed in the middle box and allowed to freely explore all three boxes for 5 min. In the social affiliation session, a strange mouse (stranger 1, gender and age-matched) was placed in either the left or right box. The experimental mouse was then allowed to freely explore all three boxes for 10 min. The movement of the mice was recorded, and the time spent near stranger 1 and the empty cage was analyzed. In the social novelty preference session, another gender and age-matched strange mouse (stranger 2) was introduced in the other cage. The test mouse was then allowed to freely explore all three chambers for another 10 min. The time spent near the familiar stranger 1 and the novel stranger 2 were recorded and compared.

### New object recognition test

This test is utilized to evaluate the recognition and memory function of the mice. The mice were placed in an open chamber (40 cm × 40 cm × 40 cm). During the adaptation stage, the mice were allowed to explore the empty chamber for 10 min. In the training session conducted 24 h later, each mouse was given the opportunity to explore two identical objects placed on a diagonal (both 10 cm from the corner) for 10 min. After four hours later (retention phase), one of the objects was replaced with a novel object that had a different shape. The mice were then allowed to freely explore for another 10 min. The preference ratio for each mouse was calculated as the percentage of time spent exploring the familiar object: Tf / (Tf + Tn) × 100%, or the novel object: Tn / (Tf + Tn) × 100%, where Tf and Tn represent the time spent exploring the familiar object and the novel object, respectively. Exploration was defined as any activity performed by the mice within 2 cm radius around the object. The facilities were cleaned with 75% ethanol between trials.

### Morris water maze test (MWM)

MWM was conducted to assess spatial learning and memory abilities between days 30–35 after CCI. During the learning phase, the mice were trained on four trials (at four fixed locations) every day between 30–34 d after CCI. The time taken by the mouse to find the platform was recorded for each trial, with a maximum time limit of 60 s. If the mouse was unable to find the platform within 1 min, the experimenter guided the mouse to find the platform, and the time was recorded as 1 min. At the end of each trial, the mouse was allowed to remain on the platform for 15 s to consolidate the spatial position of the platform. The memory test conducted on day 35 after TBI, where mice were placed in a new starting position and allowed to swim in the pool for 60 s without the platform. The swimming speed, the number of crossing platform and the duration spent in the target quadrant where the platform was previously located were recorded.

### Statistical analysis

Data analysis was performed using GraphPad Prism 8 (version 8.0, La Jolla, CA, USA), and the results were presented as mean ± SD. Prior to conducting parametric tests, the normal distribution of the data was confirmed using the Kolmogorov–Smirnov test. Two groups of data following a Gaussian distributed were compared using a two-tailed unpaired Student’s *t* test. Non-Gaussian distributed data was compared using Mann–Whitney U rank sum test. For multiple groups of data following a Gaussian distribution, ordinary one-way analysis of variance (ANOVA; F-test) was utilized, followed by Bonferroni’s multiple comparisons test. Multiple groups of data that did not follow a normal distribution were compared using the Kruskal–Wallis test. Differences among groups with repeated measurements were assessed through two-way ANOVA followed by Bonferroni’s multiple comparisons.

## Results

### Construction of ASK1-K716R.

**Fig. 1:**
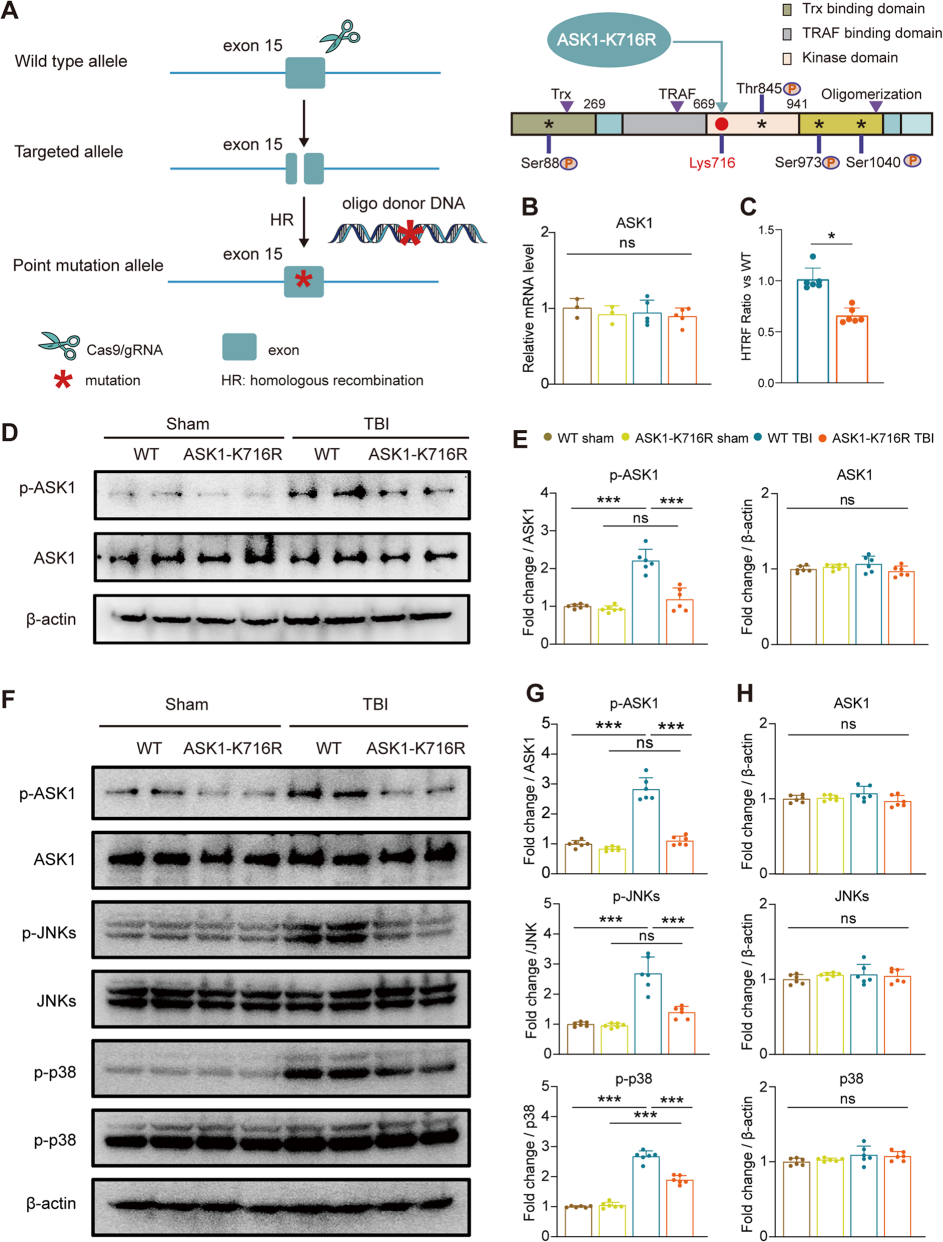
ASK1-K716R inhibits the activation of ASK1 and JNKs/p38 signaling pathway 3 days following TBI. **A** Schematic diagram depicting homologous recombination repair to introduce the ASK1 Map3k5-e (K716R) site-specific mutation. **B** Quantification of ASK1 mRNA levels in the brain using qRT-PCR. *n* = 3 for WT Sham, *n* = 3 for ASK1-K716R Sham, *n* = 5 for WT TBI, *n* = 5 for ASK1-K716R TBI. **C** Quantification of ASK1 kinase activity in TBI mice using an ASK1 kinase activity detection kit (*n* = 6/group). **D**, **E** Representative Western blot bands (**D**) and quantification (**E**) of ASK1 and p-ASK1 protein levels in the ipsilateral hemisphere 3 days following TBI (*n* = 6/group). **F–H** Representative Western blot bands (**F**) and quantification (**G**, **H**) of p-ASK1/ASK1, p-JNKs/JNKs, p-p38/p38 protein levels in the ipsilateral hemisphere 3 days following TBI (*n* = 6/group). All data from male mice are presented as the mean ± SD. One-way ANOVA test and Bonferroni post hoc (**B**, **E**, **G**, **H**) and Student’s *t* test (**C**). * *p* < *0.05,* *** *p* < *0.001,* ns, no significance, as indicated

Inhibition of ASK1 activity holds immense therapeutic potential for addressing nervous system diseases and promoting neurological function recovery following brain injury [14, 21–23]. To investigate the implications of ASK1 in the pathological progression after TBI, we generated a novel ASK1-K716R mouse model using the CRIPR/Cas9 system (Fig. 1A). DNA sequencing (Fig. S1A) confirmed the precise substitution at site 716, resulting in the translation change from lysine to arginine by altering the sequence from “AAGGAAATC” to “AGAGAAATA”. qPCR was performed to assess the impact of the ASK1-K716R mutation on ASK1 transcription. Notably, no significant difference in ASK1 mRNA expression was observed among the experimental groups (Fig. 1B), indicating that ASK1-K716R does not influence ASK1 transcription.

### ASK1-K716R suppresses phosphorylation of ASK1 and activation of JNKs/p38 signaling pathway following TBI

To investigate the impact of ASK1-K716R on ASK1 kinase activity after TBI, we measured ASK1 kinase activity using an ASK1 kinase activity detection kit. The results revealed a significant reduction in ASK1 kinase activity following TBI in ASK1-K716R TBI mice (Fig. 1C). Additionally, western blot analysis was performed 3 days post-TBI, showing no change in the overall expression of ASK1 with the mutation. However, the phosphorylation levels of ASK1 were significantly reduced in ASK1-K716R TBI mice, compared to controls (Fig. 1D, E). Furthermore, immunofluorescence staining was performed to evaluate the colocalization of p-ASK1 and (Iba1^+^) microglia, (GFAP^+^) astrocytes and (NeuN^+^) neurons, respectively (Fig. S1B). The results demonstrated that ASK1-K716R inhibited the expression of p-ASK1 in neurons, microglia, and astrocytes following TBI, supporting the effective suppression of ASK1 kinase activity by ASK1-K716R after TBI.

ASK1 is known to modulate apoptosis and inflammation by activating downstream JNKs and p38 signaling pathways [24–30]. In order to examine the impact of ASK1-K716R on the activation of JNKs/p38, western blotting was performed on day 3 post-TBI. No significant changes in the expression levels of ASK1, p38, or JNKs were observed in WT and ASK1-K716R mice, regardless of TBI (Fig. 1F, H). However, ASK1-K716R significantly inhibited the elevated expression of p-ASK1, p-JNKs, and p-p38 induced by TBI (Fig. 1F, G). Additionally, TUNEL and NeuN immunofluorescence co-staining of brain slices on day 3 post-TBI revealed a significant decrease in neuronal apoptosis in ASK1-K716R mice compared to controls (Additional file 1:Fig. S2A-C). These results indicate the effective inhibition of the ASK1/JNKs pro-apoptotic pathway by ASK1-K716R after brain injury, consequently reducing neuronal apoptosis.

### ASK1-K716R inhibits the activation of ASK1/JNKs signaling pathway in endothelial cells of cerebral microvessels

**Fig. 2:**
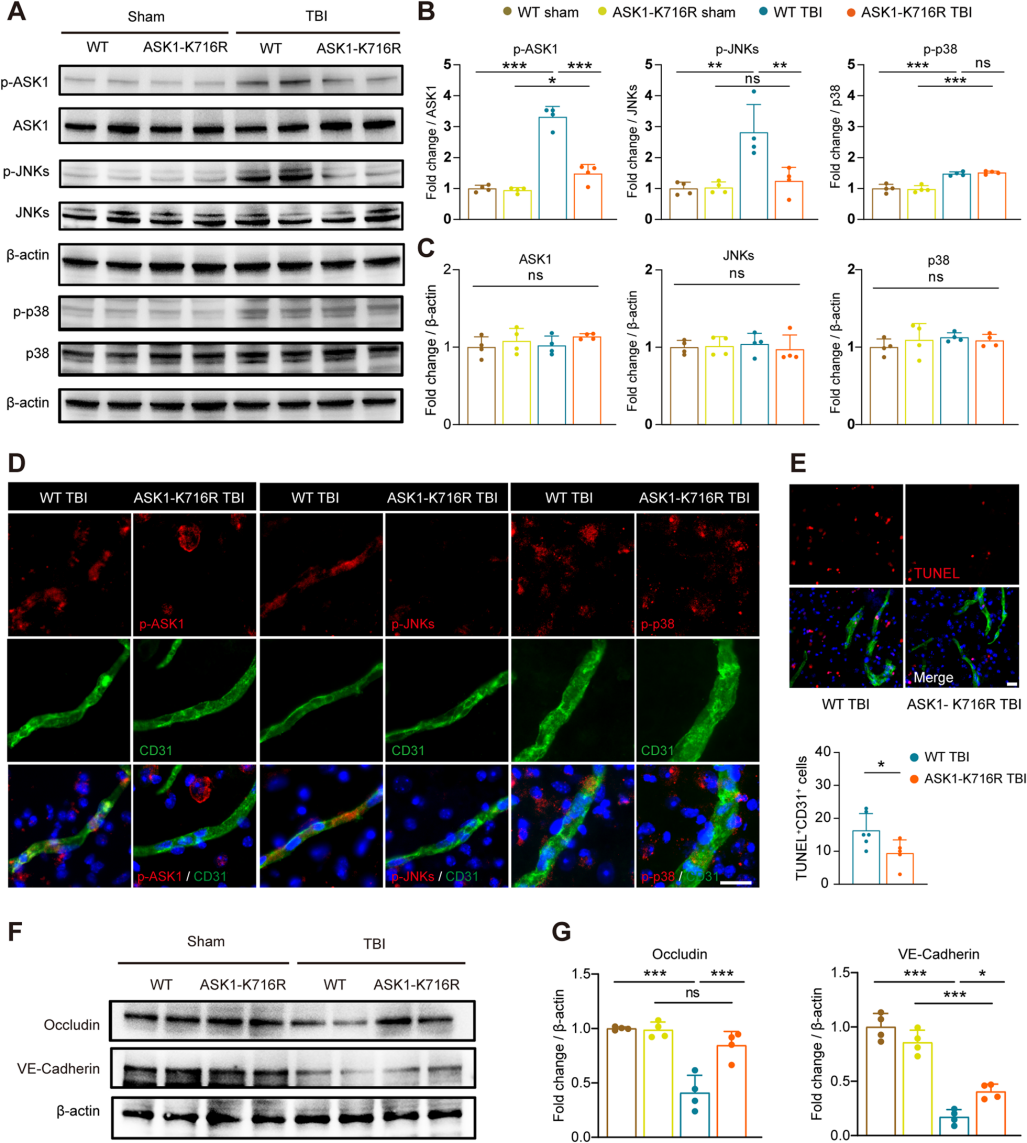
ASK1-K716R inhibits the activation of ASK1/JNKs signaling pathway and protects tight junction proteins in extracted brain microvessels. **A–C** Representative Western blot bands (**A**) and quantification (**B**, **C**) of p-ASK1/ASK1, p-JNKs/JNKs, p-p38/p38 in extracted brain microvessels (*n* = 4/group). **D** Representative images of endothelial cells (CD31^+^) expressing p-ASK1, p-JNKs, and p-p38 at peri-lesion cortex 3 days following TBI. Scale bar, 20 μm. **E** Representative images and quantification of TUNEL^+^ (red) and CD31^+^ (green) staining at peri-lesion cortex 3 days following TBI. *n* = 6 for WT TBI, *n* = 5 for ASK1-K716R TBI. Scale bar, 20 μm. **F**, **G** Representative Western blot bands (**F**) and quantification (**G**) of Occludin and VE-Cadherin in extracted brain microvessels. *n* = 4/group. All data from male mice are presented as the mean ± SD. One-way ANOVA test and Bonferroni post hoc (**B**, **C**, **F**, **G**) and Student’s *t* test (**E**). * *p* < 0.05, ** *p* < 0.01, *** *p* < 0.001, ns, no significance, as indicated

Elevated phosphorylation of ASK1 has been associated with endothelial inflammation, apoptosis, and oxidative stress [31, 32]. Therefore, to explore the potential impact of ASK1 signaling pathway on BBB integrity following TBI, cerebral microvessels were isolated from the injured cerebral hemisphere of TBI mice. The protein expression of ASK1/p38/JNKs signaling pathway was quantitatively analyzed using Western blot analysis. The results showed no significant changes in the expression of ASK1, p38, or JNKs in cerebral microvessels from both WT and ASK1-K716R mice, regardless of TBI (Fig. 2A, C). However, ASK1-K716R significantly reduced the phosphorylation levels of ASK1 and JNKs in cerebral microvessels following TBI. Interestingly, unlike the findings in brain tissue samples, ASK1-K716R did not alter the phosphorylation level of p38 in cerebral microvessels after TBI (Fig. 2A, B). These results were further supported by double staining of CD31 with p-ASK1, p-JNKs, and p-p38 (Fig. 2D). Considering the involvement of ASK1/JNKs axis in apoptosis, we performed TUNEL staining to evaluate endothelial apoptosis following TBI. Our results demonstrated a reduction in the number of TUNEL^+^ CD31^+^ microvessels in the ASK1-K716R TBI group compared to the WT TBI group (Fig. 2E), further confirming the effective inhibition of ASK1 signaling by ASK1-K716R after brain injury and its subsequent reduction in endothelial apoptosis.

Inhibition of ASK1 has been shown to stabilize endothelial tight junctions, thus ameliorating endothelial barrier dysfunction [33–36]. Additionally, we evaluated the expression of tight junction proteins in cerebral microvessels using Western blotting and immunofluorescence staining. The data indicated that ASK1-K716R alleviated the compromised expression of Occludin and vascular endothelial cadherin (VE-Cadherin) induced by TBI (Fig. 2F, G, Additional file 1: Fig. S3A-B).

### ASK1-K716R attenuates BBB permeability and preserves BBB integrity

**Fig. 3:**
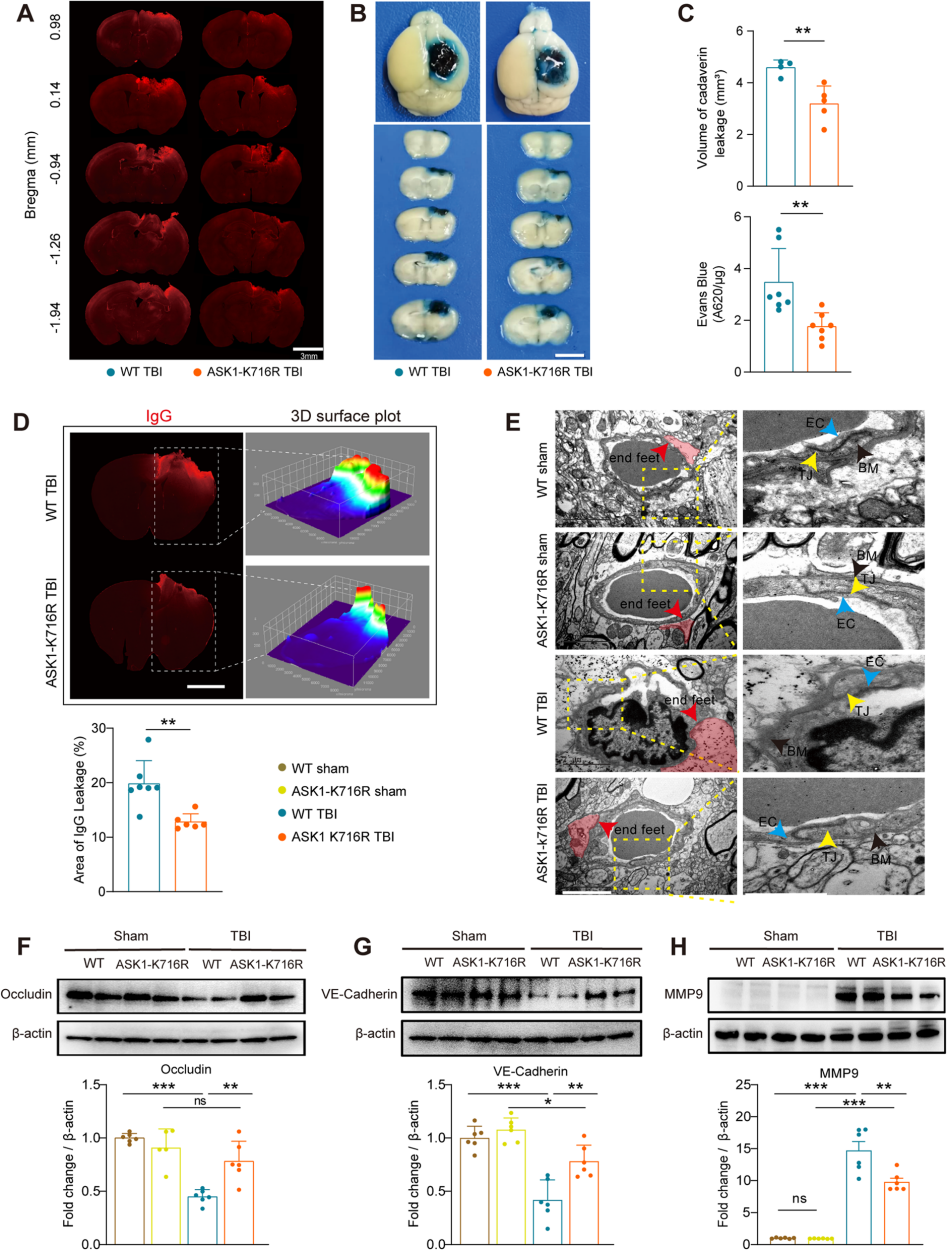
ASK1-K716R ameliorates BBB disruption 3 days following TBI. **A** Representative images of extravasation of Alexa 555 cadaverine. Scale bar, 3 mm. **B** Representative images of extravasation of Evans blue. Scale bar, 1 cm. **C** Volume of the brain with leakage of cadaverine (upper panel, *n* = 4 for WT TBI, *n* = 5 for ASK1-K716R TBI) and the quantification of extravasation Evans blue (lower panel, *n* = 7/group). **D** Representative images and 3D surface plot of IgG leakage with quantification analysis. *n* = 7 for WT TBI, *n* = 6 for ASK1-K716R TBI. **E** Representative transmission electron microscope images of BBB structure. Scale bar, 2 μm. Red arrows indicate swollen astrocyte end-feet. Black arrows indicate the basement membrane. Blue arrows indicate endothelial cells. And yellow arrows indicate tight junctions between endothelial cells. **F**–**H** Representative Western blot bands and quantification of Occludin (**F**), VE-Cadherin (**G**), and MMP 9 (**H**) in the ipsilateral hemisphere 3 days following TBI (*n* = 6/group). All data from male mice are presented as the mean ± SD. One-way ANOVA test and Bonferroni post hoc (**F**–**H**) and Student’s *t* test (**C**). * *p* < 0.05, ** *p < 0.01,* *** *p* < 0.001, ns, no significance, as indicated

To investigate the potential protective role of ASK1-K716R in preserving BBB function by modulating endothelial apoptosis, we assessed BBB integrity on day 3 post-TBI by evaluating the central permeation of peripherally injected tracers, including fluorescent Alexa 555 cadaverine and EB. Analysis of cadaverine fluorescence intensity revealed a decrease in leakage volume in the ASK1-K716R TBI group compared to the WT TBI group (Fig. 3A, C upper panel). In the EB assay, extensive leakage of EB was observed in the ipsilateral hemispheres of WT TBI mice (Fig. 3B), while EB extravasation was significantly reduced in ASK1-K716R TBI mice, as evident both visually and quantitatively (Fig. 3B, C lower panel). Concurrently, immunofluorescence staining for IgG was performed to examine endogenous peripheral IgG leakage into the brain parenchyma, and a notable reduction in IgG leakage was observed in the coronal brain sections of ASK1-K716R TBI mice compared to WT TBI mice (Fig. 3D). Furthermore, transmission electron microscopy of BBB ultrastructure revealed characteristics of BBB damage in WT mice following TBI, including swollen astrocyte endfeet, thickened basement membranes, and decreased expression of endothelial tight junction protein. Conversely, ASK1-K716R ameliorated these impairments (Fig. 3E). The restoration of BBB integrity was further confirmed by western blotting analysis, which showed that the downregulation of tight junction proteins, such as Occludin and VE-Cadherin, induced by TBI was significantly alleviated by ASK1-K716R (Fig. 3F, G). It has been reported that MMP-9, originating from various cell sources, contributes to the degradation of tight junction proteins in brain injury [37–39]. Consistent with this, western blotting revealed an upregulation of MMP-9 in WT-TBI mice, whereas ASK1-K716R prominently suppressed MMP-9 expression after TBI (Fig. 3H). Collectively, these findings demonstrate that ASK1-K716R reduces BBB permeability and safeguards its integrity following TBI.

### ASK1-K716R inhibits the infiltration of peripheral immune cells and microglia-mediated neuroinflammation following TBI

**Fig. 4:**
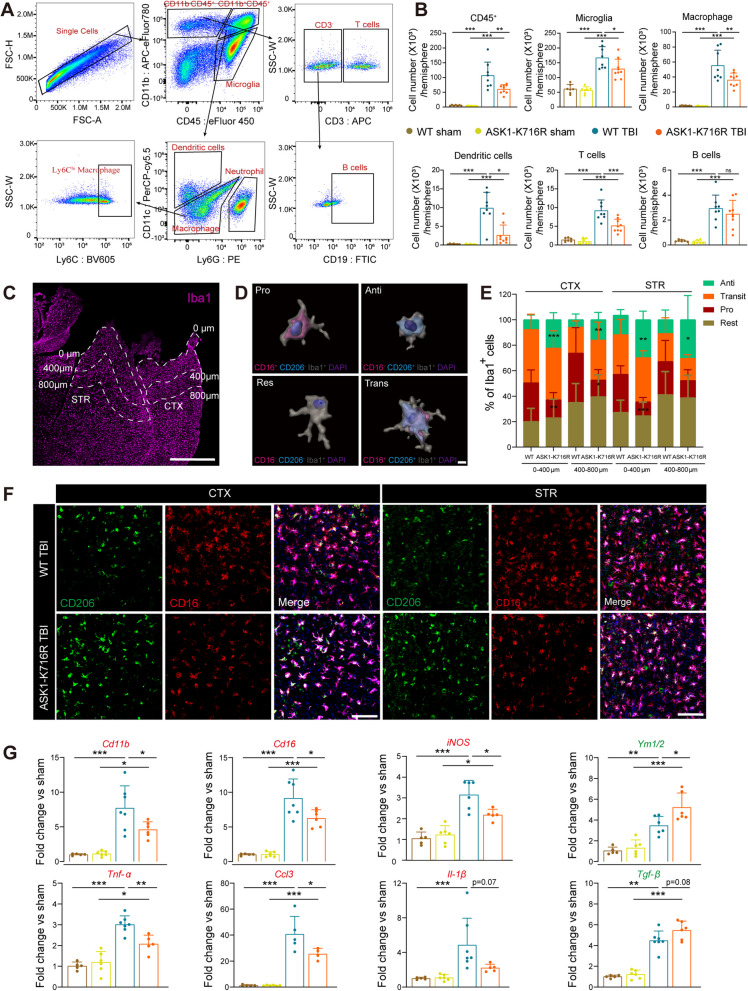
ASK1-K716R inhibits neuroinflammation and the infiltration of peripheral immune cells 3 days after TBI. **A** Illustration of flow cytometry analysis of circulating immune cells in the brain 3 days following TBI. **B** Quantification of the numbers of infiltrating immune cells in the brain. *n* = 7 for WT Sham, *n* = 7 for ASK1-K716R Sham, *n* = 8 for WT TBI, *n* = 9 for ASK1-K716R TBI. **C** Schematic diagram showing the regions from the damaged edge proximal (0–400 µm) and distal (400–800 µm) of the CTX or STR. Scale bar, 1 mm. **D** 3D reconstruction of four phenotypes of microglia/macrophages: pro- (CD16^+^CD206^−^Iba1^+^), anti- (CD16^−^CD206^+^Iba1^+^), transit- (CD16^+^CD206^+^Iba1^+^), and rest- (CD16^−^CD206^−^Iba1^+^) microglia/macrophages. **E** Quantification of the percentage of four phenotypes in all Iba1^+^ microglia/macrophages in the 0–400 µm or 400–800 µm from the damaged edge in CTX and STR 3 days following TBI. *n* = 6/group. **F** Representative images of immunostaining for microglia/macrophages in the CTX or STR 3 days following TBI. Scale bar, 100 μm. **G** Measurement of a panel of inflammation markers by qRT-PCR in the ipsilateral hemisphere 3 days following TBI. *n* = 5 for WT Sham, *n* = 5 for ASK1-K716R Sham, *n* = 7 for WT TBI, *n* = 5 foe ASK1-K716R TBI. All data from male mice are presented as the mean ± SD. One-way ANOVA test and Bonferroni post hoc (**B**, **G**) and Student’s *t* test (**E**). * *p* < 0.05, ** *p* < 0.01, *** *p* < 0.001, ns, no significance, ASK1-K716R TBI *vs.* WT TBI, or as indicated

Circulating leukocytes that breach the compromised BBB play a pivotal role in the aftermath of TBI. The substantial influx of leukocytes is a primary contributor to the surge of MMPs during the early stage of brain injury [40]. We employed a flow cytometry-based approach to quantitatively assess the infiltration of peripheral immune cells in the brains on day 3 after TBI (Fig. 4A). The findings revealed no significant variation in cell numbers between the two Sham groups, suggesting that ASK1-K716R does not exert an influence on brain immune cells under physiological conditions (Fig. 4B). Notably, the numbers of peripheral infiltrating immune cells (CD45^hi^CD11b^+^), dendritic cells (CD11b^+^CD45^+^CD11c^+^Ly6G^−^), macrophages (CD45^hi^CD11b^+^CD11c^−^Ly6G^−^), T cells (CD11b^−^CD45^+^CD3^+^), and B cells (CD11b^−^CD45^+^CD3^−^CD19^+^) were observed to increase after TBI. However, ASK1-K716R significantly attenuated these increases, except for B cells (Fig. 4B).

To investigate the impact of ASK1-K716R on peripheral immune cell levels following TBI, we examined the changes in various immune cell populations in peripheral blood and spleen using flow cytometry 3 days post-TBI. In both peripheral blood and spleen, the results revealed no significant differences in populations of macrophages, dendritic cells, Ly6C^high^ inflammatory macrophages, T cells and B cells (Fig. S4A-C). These results further support the notion that ASK1-K716R does not alter the levels of immune cells in peripheral blood and spleen. However, it primarily inhibits the infiltration of various immune cells into the brain following TBI.

The ASK1-K716R variant’s protective effect on BBB dysfunction may reduce microglia-mediated neuroinflammation following TBI. To evaluate the heterogeneity of microglia/macrophages on day 3 after TBI, we employed triple immunofluorescence staining with Iba1 (a marker for microglia/macrophage), CD16 (a marker for pro-inflammatory phenotype), and CD206 (a marker for anti-inflammatory phenotype) to categorize the microglia/macrophages into four distinct phenotypes (Fig. 4D): pro-inflammatory (CD16^+^CD206^−^Iba1^+^, Pro), transient (CD16^+^CD206^+^Iba1^+^, Trans), anti-inflammatory (CD16^−^CD206^+^Iba1^+^, Anti), and resting (CD16^−^CD206^−^Iba1^+^, Rest) microglia/macrophages. The results demonstrated that TBI induced the activation of microglia/macrophages in the peri-lesion area, while ASK1-K716R didn’t alter the activation of microglia/macrophages, as indicated by the similar proportion of resting microglia/macrophages between ASK1-K716R TBI and WT TBI groups. Importantly, ASK1-K716R significantly decreased the proportion of the Pro phenotype and increased the proportion of Anti phenotype in the peri-lesion area located 0–400 µm and/or 400–800 µm from damaged edge of the cortex and striatum. However, there was no significant change observed in the Trans phenotype within the area located 0–400 µm and/or 400–800 µm from damaged edge of the cortex and striatum, 3 days after TBI (Fig. 4C–F). Overall, these results suggest the involvement of ASK1-K716R in the regulation of microglia/macrophage heterogenization following TBI.

The infiltration of peripheral immune cells and alterations in microglia heterogeneity have a substantial impact on the inflammatory environment within the brain. To further investigate the impact of ASK1-K716R on the expression of inflammatory mediators, we performed qPCR analysis 3 days after TBI. Our findings revealed a significant upregulation of pro-inflammatory factors (*Cd11b,* *Cd16,* *iNOS,* *Tnf-α,* *Il-1β,* and *Ccl3*) as well as anti-inflammatory factors (*Ym1/2,* *Tgf-β*) following TBI (Fig. 4G). ASK1-K716R inhibited the expression of several pro-inflammatory factors, specifically *Cd11b,* *Cd16,* *iNOS,* *Tnf-α* *and* *Ccl3*, while concurrently enhancing the expression of an anti-inflammatory factor *Ym1/2* (Fig. 4G). As a result, ASK1-K716R primarily inhibits the pro-inflammatory response and stimulates the anti-inflammatory response, consequently ameliorating the inflammatory microenvironment within the brain.

### ASK1-K716R attenuates white matter injury (WMI) following TBI

**Fig. 5:**
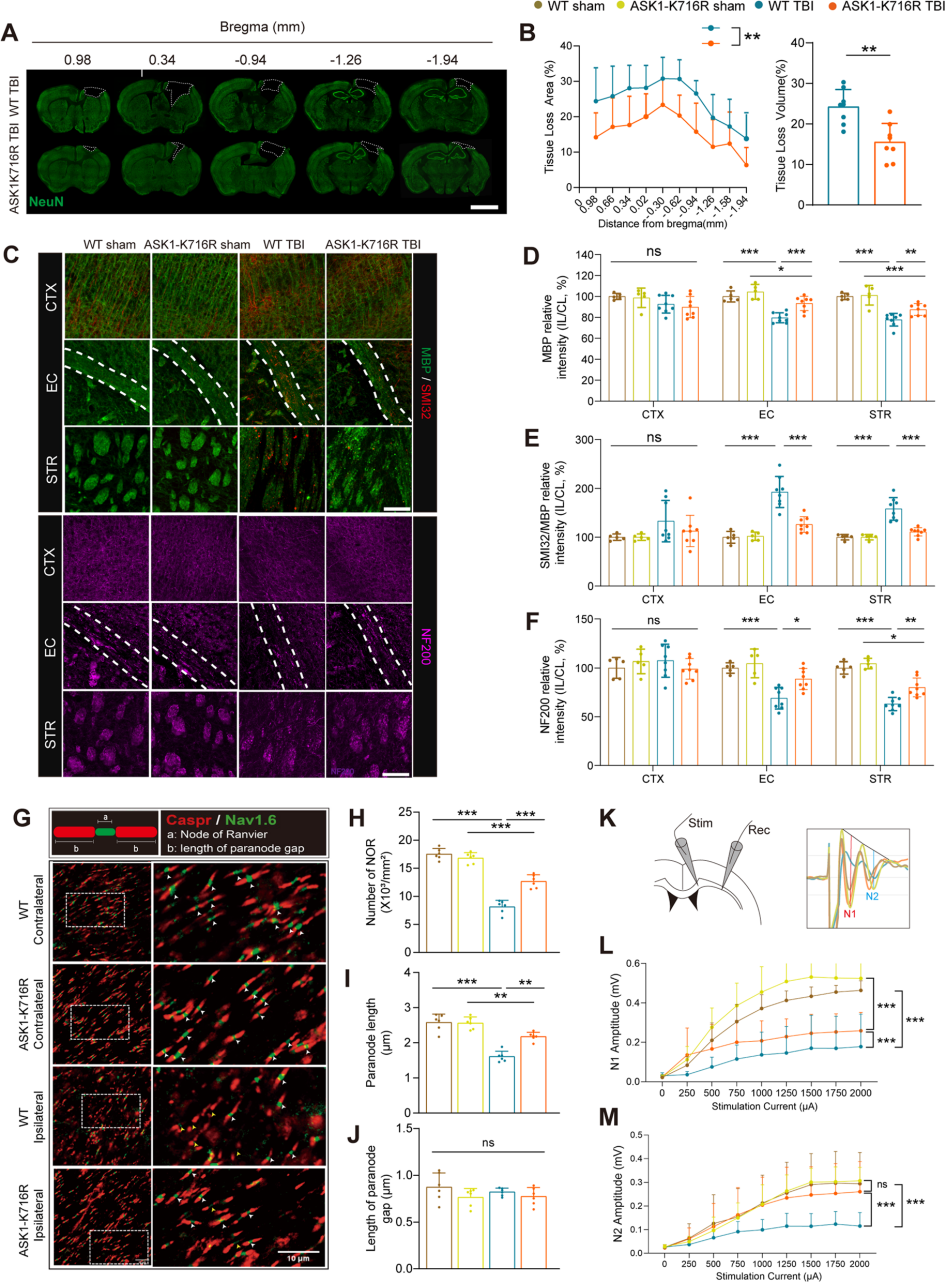
ASK1-K716R improves brain tissue loss and white matter integrity 35 days following TBI. **A** Representative images of NeuN^+^ staining. Scale bar, 3 mm. **B** Quantification of tissue loss area and tissue loss volume. *n* = 8/group. **C** Representative images of MBP (green), SMI32 (red), and NF200 (purple) immunostaining in the CTX, EC, and STR 35 days following TBI. Scale bar, 100 μm. **D**–**F** Quantification of the fluorescence intensity of MBP, SMI32/MBP, and NF200 in CTX, EC, and STR 35 days following TBI. *n* = 5 for WT Sham, *n* = 5 for ASK1-K716R Sham, *n* = 8 for WT TBI, *n* = 8 for ASK1-K716R TBI. **G** Representative images of the NOR with Caspr (red) and Nav1.6 (green) immunostaining at the corpus callosum 35 days following TBI. Scale bar, 10 μm. **H**–**J** Quantification of the number of NOR (**I**), length of paranodes (**J**), and length of paranode gap (**K**). *n* = 6/group. **L** Schematic diagram of illustrating the placement of the stimulus electrode, record electrode, as well as representative CAPs. **L**, **M** Quantification of N1 **(L)** and N2 **(M)** amplitudes under different stimulation currents ranging from 0 to 2000 μA. *n* = 7 for WT Sham, *n* = 7 for ASK1-K716R Sham, *n* = 10 for WT TBI, and *n* = 10 for ASK1-K716R TBI. All data from male mice are presented as the mean ± SD. One-way ANOVA test and Bonferroni (**D**–**F**, **H**–**J**, **L**, **M**) post hoc and Student’s *t* test (**B**). * *p* < *0.05,* ** *p* < *0.01,* *** *p* < *0.001,* ns, no significance, as indicated

It is of interest to determine whether ASK1-K716R can have a sustained protective effect on TBI by preserving BBB integrity and suppressing inflammatory responses in the brain during the acute phase. To assess long-term tissue loss, immunohistochemical staining of NeuN was used as a standard method for estimating TBI (Fig. 5A). Our findings revealed that, on day 35 following TBI, ASK1-K716R significantly mitigated the extent and volume of neuronal tissue loss compared to WT TBI mice (Fig. 5B).

WMI is a significant pathological consequence in the context of TBI, playing a crucial role in the pathophysiological processes [41, 42]. WMI, characterized by demyelination and/or axonal damage, has been closely linked to neuroinflammation following acute or chronic brain injury. In this study, we employed immunofluorescence co-staining of MBP/SMI32 and NF200 to label myelin and neurofilaments, respectively, on day 3 and 35 post-TBI (Fig. 5 C, Additional file 1: Fig. S5A-C). Our findings demonstrated that traumatic injury resulted in a reduction in MBP fluorescence intensity and an increase in the relative fluorescence intensity of SMI32/MBP within the external capsule (EC) and striatum. Notably, ASK1-K716R treatment effectively ameliorated TBI-induced demyelination on both days 3 and 35 post-TBI (Fig. 5D–E, Additional file 1: Fig. S5D-E). Furthermore, the neuroprotective effect of ASK1-K716R against axonal injury was evident through NF200 staining (Fig. 5F, Additional file 1: Fig. S5 F). Nodes of Ranvier (NOR) are essential for signal conduction and represent unmyelinated segments between two myelin sheaths in myelinated nerve fibers [43]. Immunofluorescence staining for contactin-associated protein (Caspr) and sodium channel (Nav1.6) was performed to evaluate NOR in the corpus callosum (CC) 35 days after TBI (Fig. 5G). We observed a significant reduction in the number of NOR and paranode length following TBI compared to the contralateral region. Notably, ASK1-K716R remarkably alleviated these impairments in comparison to the WT TBI group (Fig. 5H-J). Additionally, we measured the fast potential of myelinated axon conduction (N1) and the slow potential of unmyelinated axon conduction (N2) in the CC to assess the conductive capacity of white matter fibers (Fig. 5K). No significant difference was observed between the two Sham groups, indicating that ASK1-K716R did not affect nerve conduction under physiological conditions. TBI caused a decrease in both N1 and N2 potential amplitudes in WT mice. In contrast, ASK1-K716R TBI mice exhibited a mild improvement in the N1 amplitude and a notable recovery of the N2 amplitude to levels comparable to the control group (Fig. 5L, M). These findings suggest that ASK1-K716R has a potent neuroprotective effect against brain tissue loss and white matter injury, holding promise as a potential therapeutic target for TBI management.

### ASK1-K716R ameliorates long-term neurological deficits following TBI

**Fig. 6:**
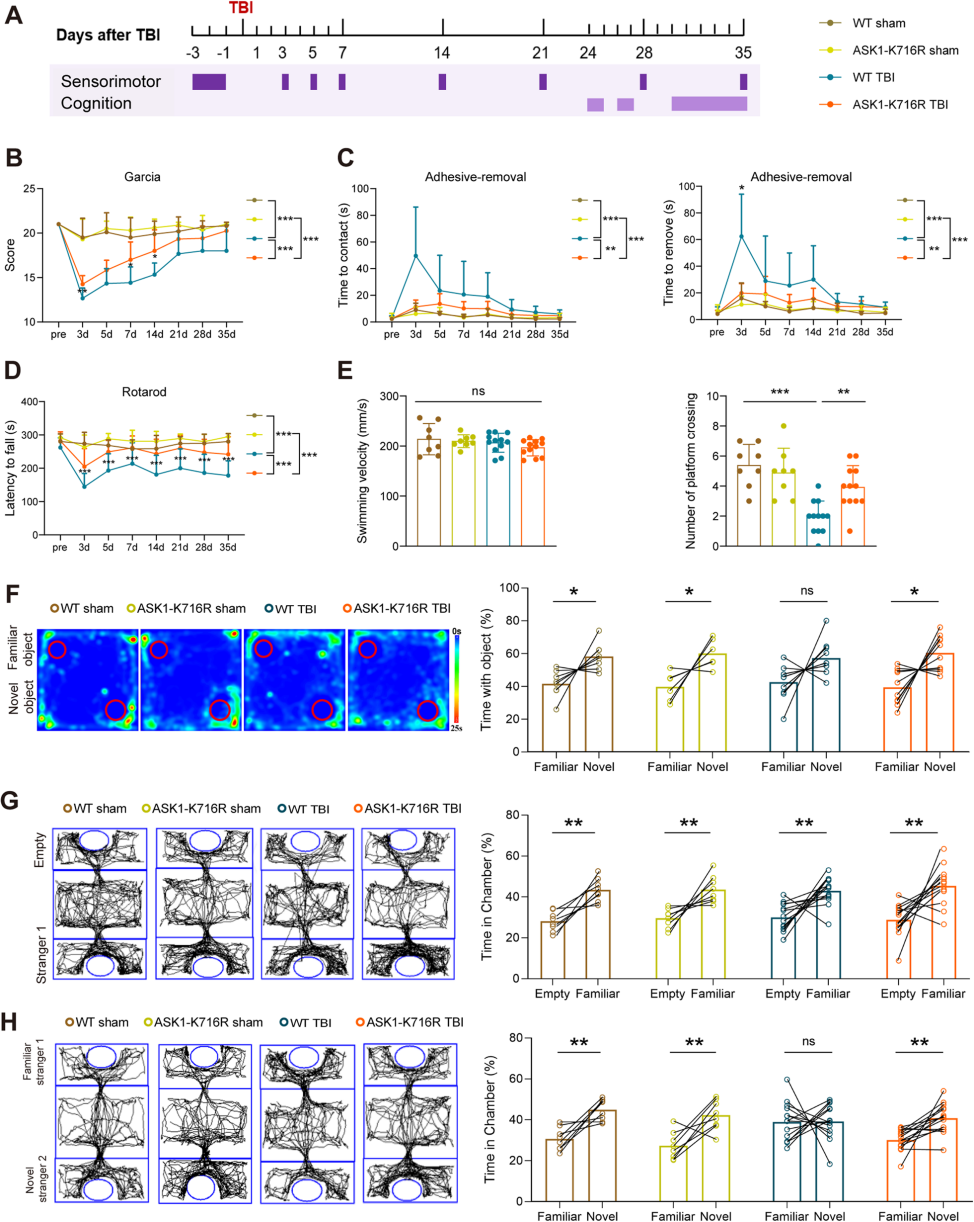
ASK1-K716R ameliorates long-term neurological dysfunction following TBI. **A** The timeline of behavioral tests is depicted in the diagram. **B** Score of the modified Garcia score test. *n* = 10 for WT Sham, *n* = 10 for ASK1-K716R Sham, *n* = 12 for WT TBI, *n* = 12 for ASK1-K716R TBI. **C** Time taken to contact and remove the tape in the adhesive removal test. *n* = 6 for WT Sham, *n* = 6 for ASK1-K716R Sham, *n* = 10 for WT TBI, *n* = 10 for ASK1-K716R TBI. **D** Latency to fall in the rotarod test. *n* = 8 for WT Sham, *n* = 8 for ASK1-K716R Sham, *n* = 14 for WT TBI, *n* = 14 for ASK1-K716R TBI. **E** Spatial learning and memory assessed in the Morris Water Maze, including the time spent in the target quadrant and swimming velocity (34 d after TBI). *n* = 8 for WT sham, *n* = 8 ASK1-K716R sham, *n* = 12 WT TBI, *n* = 12 ASK1-K716R TBI. **F** Representative track heat maps and time spent exploring objects in the new object recognition test. *n* = 8 for WT Sham, *n* =6 for ASK1-K716R Sham, *n* = 10 for WT TBI, and *n* = 10 for ASK1-K716R TBI. **G**, **H** Representative traces and time spent interacting with empty cages, stranger mouse 1, and stranger mouse 2 in the social affiliation session (**G**) and the social novelty preference session (**H**). *n* = 8 for WT Sham, *n* = 8 for ASK1-K716R Sham, *n* = 10 for WT TBI, *n* = 10 for ASK1-K716R-TBI. All data from male mice are presented as the mean ± SD. Two-way ANOVA repeated measurement (**B**–**E**) and paired Student’s *t* test (**F**–**H**, right). * *p* < 0.05, ** *p* < 0.01, *** *p* < 0.001, ns, no significance, as indicated

Previous studies have demonstrated a significant association between white matter injury and behavioral changes [44, 45]. To evaluate the impact of ASK1-K716R on sensorimotor dysfunction induced by TBI, we performed a battery of behavioral tests, including the modified Garcia score test, rotarod test, and adhesive-removal test from 3 to 35 days post-CCI (Fig. 6A). Remarkably, mice in the ASK1-K716R Sham group exhibited no neurological deficits in multiple behavioral tests compared to WT Sham mice (Fig. 6B–E). Particularly noteworthy, the ASK1-K716R TBI group displayed a significantly faster improvement in the modified Garcia score compared to the WT TBI group (Fig. 6B). Furthermore, the WT-TBI group mice exhibited a shorter latency to fall off in the rotarod test (Fig. 6D) and a prolonged latency to touch and remove sticky paper in the adhesive removal test, in comparison to the ASK1-K716R-TBI group mice (Fig. 6C). The results of these behavioral tests indicate that ASK1-K716R significantly improved TBI-induced sensorimotor deficits without affecting neurological function in the Sham groups.

To investigate the effect of ASK1-K716R on cognitive abilities after TBI, we performed three behavioral tests, including the new object recognition test, three-chamber social test, and MWM test within 25–35 days post-TBI. The new object recognition test aimed to evaluate recognition memory based on the mice’s preference for exploring novel objects. Remarkably, WT TBI mice showed no variation in their exploration tendencies toward familiar or new objects, while ASK1-K716R TBI mice displayed a restored preference for novel objects, indicating an improvement in recognition memory deficit (Fig. 6F). The cognitive improvements resulting from ASK1-K716R were further confirmed in the three-chamber social test, which measures social novelty recognition ability. During the adaptation period, both WT and ASK1-K716R mice exhibited normal sociability, interacting frequently with other mice rather than empty cages (Fig. 6G). However, the decline in preference for novel mice observed in the WT TBI group was mitigated in the ASK1-K716R-TBI group, highlighting the potential of ASK1-K716R to alleviate the impairment in social novelty recognition (Fig. 6H). Furthermore, we evaluated spatial cognitive function using the Morris Water Maze (MWM test at 30–35 days after CCI. Both TBI groups showed prolonged escape latency during the training period, ASK1-K716R did not influence the spatial learning decline in TBI mice (Additional file 1: Fig. S6A upper panel). However, during the probe test, ASK1-K716R attenuated the decrease in the number of platform crossings, with no difference in swimming velocity, induced by TBI (Fig. 6E, Additional file 1: Fig. S6A lower panel). Collectively, these behavioral findings demonstrate the beneficial effects of ASK1-K716R on social novelty recognition and memory restoration after TBI.

## Discussion

Targeted silencing ASK1 has been suggested to have a potential neuroprotective effect [13, 14]. In this study, we employed the CRIPR/Cas9 system to induce a site-specific mutation (ASK1 Map3k5-e K716R) in mice, resulting in the inhibition of ASK1 phosphorylation following TBI. ASK1-K716R was found to decrease endothelial cell apoptosis and prevent tight junction protein degradation by suppressing ASK1/JNKs signaling pathway, thereby improving the integrity of BBB after TBI. Additionally, ASK1-K716R effectively inhibited the infiltration of peripheral immune cells and ameliorated neuroinflammation after TBI by modulating the pro-inflammatory phenotype of microglia/macrophages towards an anti-inflammatory phenotype. Histological experiments further demonstrated that ASK1-K716R reduced TBI-induced pathological damage to the neurons, white matter, and NOR in ASK1-K716R mice. Furthermore, compared to WT mice, ASK1-K716R mice subjected to TBI exhibited alleviated neurological deficits and improved social novelty recognition and memory.

ASK1 activity can be regulated by various post-translational modifications, and its phosphorylation at different sites can lead to varying effects on its activity [46–54]. The KD of ASK1 is critical for its activity region and represents a potential target site for developing targeted inhibitor drugs. Lysine 716, located in the ATP binding of ASK1-KD, is crucial for its kinase activity. Since the ATP binding site of ASK1-KD is located in Lys716, it represents a potential target site for regulating ASK1 activity. In this study, we aimed to generate a mutant model of Lys716 to reduce the activity of ASK1 and investigate neuroprotective effect. To this end, we introduced a site-specific mutation (K716R) in the ASK1 gene using the CRISPR/Cas9 system. The mutation specifically regulates the phosphorylation level of ASK1 while having no effect on protein expression levels. Our findings demonstrated that ASK1-K716R effectively reduced ASK1 phosphorylation in all cells and throughout the body after TBI. This inhibition of ASK1 activity through the K716R mutation holds promise as a strategy to improve the neurological function in TBI patients. Furthermore, the interaction between ASK1-KD and 14-3-3 protein plays a crucial role in the regulation of ASK1 activity. Specifically, the binding of 14-3-3 protein to the active site of ASK1-KD may impede accessibility to the active site and potentially affect its conformation [55]. Future studies using ASK1 site-directed mutants can further investigate the impact of ASK1m on the interaction between 14–3-3 protein and ASK1-KD through CO-IP, thereby shedding light on how ASK1-K716R affects active site accessibility and conformational changes.

ASK1 and its downstream molecules, JNKs, are critical regulators of the physiological and pathological processes of endothelial cells [56]. Inhibition of ASK1 and JNKs, both pharmacologically and genetically, can prevent the degradation of endothelial tight junction proteins and preserve BBB permeability in pathological conditions [34, 36]. Previous studies have shown that modulation of the ASK1/JNKs pathway can attenuate endothelial inflammation, reduce oxidative stress, and inhibit apoptosis under challenging conditions [31, 32, 35]. Consistent with these findings, we observed a decrease in the phosphorylation of ASK1 and JNKs in the endothelial cells of ASK1-K716R TBI mice, indicating that ASK1-K716R exerts a protective effect against TBI-induced BBB disruption by inhibiting the ASK1/JNKs pathway. Interestingly, observed that ASK1-K716R significantly reduced the activation of p38 throughout the whole brain. However, this effect was not mirrored in the activation of p38 in the cerebral microvessels. It’s worth noting that ASK1 is not the exclusive upstream regulator of p38. Other upstream kinases, such as transforming growth factor-β-activated protein kinase 1 (TAK1) and mixed lineage kinase (MLK) -2/3, can also trigger p38 activation [57, 58]. For instance, TAK1 binding protein 1 (TAB1) interacts with p38 to form TRAF6-TAB1-p38α, ultimately leading to p38α autophosphorylation and activation [59]. Therefore, we speculate that post-TBI microvessels may involve additional signal molecules beyond ASK1 in regulating p38 phosphorylation. This hypothesis warrants further exploration and verification.

In our study, compared to their WT counterparts, ASK1-K716R mice showed reduced BBB leakage as evidenced by tracer injection into the femoral vein and endogenous IgG labeling. Additionally, ASK1-K716R TBI mice exhibited up-regulated expression of tight junction proteins, namely Occludin and VE-Cadherin, providing a mechanistic basis for the protective role of ASK1-K716R in preserving the structural and functional integrity of BBB. BBB breakdown, as one of the crucial pathophysiological changes induced by TBI, contributes to serum proteins leakage, immune cells infiltration, and perivascular inflammation, ultimately exacerbating secondary injury and neurological dysfunction.

Interestingly, we observed that ASK1-K716R significantly reduced p38 activation in brain parenchyma, however, it had no effect on the phosphorylation level of p38 in cerebral microvessels following TBI. Furthermore, immunofluorescence staining revealed phosphorylated ASK1 in microglia after TBI. Compared to WT TBI mice, ASK1-K716R TBI mice exhibited significantly reduced phosphorylation levels of ASK1 and p38, accompanied by a shift in microglial polarization towards an M2 phenotype and a decrease in infiltrated peripheral macrophages. Previous studies have emphasized the importance of the ASK1-p38 pathway in microglial polarization and neuroinflammation [25–29]. Suppression of ASK1 has been shown to reduce the phosphorylation of JNKs and p38, as well as the release of pro-inflammatory factors by activated microglia [28, 29]. During the process of polarization, reactive microglia/macrophages switch between pro- and anti-inflammatory phenotypes depending on the inflammatory milieu. This dynamic polarization leads to the secretion of pro-inflammatory cytokines (such as *IL-1*, *IL-12*, and *IL-18*) to exacerbate histological damage or the secretion of anti-inflammatory regulators (such as *IL-4*, *IL-10*, and *IL-13*) to promote histological repair [60, 61]. In our study, administration of ASK1-K716R inhibited the expression of several pro-inflammatory factors, including *CD11b*, *CD16*, *iNOS*, *TNF-α* and *CCL3*, while upregulating the expression of an anti-inflammatory factor-*YM1/2*. These findings suggest a potential connection between the ASK1/p38 pathway and the pro-inflammatory reaction of microglia, rather than endothelial cells following TBI. However, the precise role of ASK1 in microglia/macrophages and whether it can alleviate neuroinflammation by inhibiting p38 activation require further investigation. In the future research, we plan to explore the specific role of ASK1 on microglia/macrophages activation after TBI using mice with microglia-specific ASK1 knock-out or mutation. Additionally, we will investigate the targeting mechanism of ASK1 inhibition on microglia/macrophages in vivo and in vitro by interfering with ASK1 activity through the use of ASK1 inhibitors or siRNA.

The early inflammatory response following TBI contributes to neural microenvironment homeostasis, however, prolonged and excessive neuroinflammation can lead to long-term tissue damage and impaired functional outcomes [61–66]. In this study, immunofluorescence staining revealed fewer neurons co-localizing with phosphorylated ASK1 in ASK1-K716R TBI mice compared to WT TBI mice, indicating an increase in neuronal survival and reduced gray matter loss in the ASK1-K716R TBI group. Previous studies have highlighted the vulnerability of white matter to cerebral ischemia and subtle changes in the brain’s extracellular microenvironment [67, 68]. TBI can result in persistent white matter injury, including axonal injury and myelinic degeneration, thereby contributing to motor, cognitive, and psychiatric deficits [69–71]. Given that ASK1 activation promotes cell death under stress, targeting ASK1 inhibition has been explored as a potential therapeutic approach to mitigate neuronal apoptosis and pyroptosis in various neurological diseases [72–74]. ASK1^−/−^ mice exhibited resistance to white matter lesions induced by chronic cerebral hypoperfusion [22] and demyelination in experimental autoimmune encephalomyelitis [25]. Similarly, in our study, we observed improved TBI-induced demyelination and axonal injury on days 35 after TBI in ASK1-K716R treated mice. Besides, transmission electron microscope and CAPs recording provided evidence of functional improvement of white matter. Finally, a battery of behavior tests demonstrated the long-term neuroprotective effects of ASK1-K716R.

## Conclusion

ASK1-K716R preserves BBB integrity by inhibiting ASK1/JNKs pathway in endothelial cells, and alleviates early neuroinflammation by modulating the polarization of microglia/macrophages. This leads to a reduction in white matter injury and ultimately promotes the recovery of neurological function following TBI.

### Supplementary Information

**Additional** **file** **1:** **Table** **S1.** Primer sequences used in real-time PCR analysis. **Fig.** **S1.** Partial DNA sequencing after ASK1-K716R and p-ASK1 expressed in brain cells. A A schematic representation of the nucleotide substitution (-AAGGAAATC- to -AGAGAAATA-) at site 716, resulting in the replacement of lysine with arginine during translation. B Representative immunofluorescence images displaying the colocalization of microglia (Iba1^+^), astrocytes (GFAP^+^), and neurons (NeuN^+^) with p-ASK1 in cortex of both WT TBI and ASK1-K716R TBI mice. Scale bar, 50 μm. **Fig.** **S2.** Effect of ASK1-K716R on neuronal apoptosis at day 3 post-TBI. A A schematic diagram showing the regions of interest around the lesioned site. Scale bar, 50 μm. The rectangle illustrates where images were taken. B Representative images of TUNEL staining in the cortical region around the lesioned site. C Quantification of the number of TUNEL^+^ neurons per hemisphere (*n* = 5 WT sham, *n* = 6 ASK1-K716R sham, *n* = 6 WT TBI, *n* = 6 ASK1-K716R TBI). All data from male mice are presented as the mean ± SD. One-way ANOVA test and Bonferroni post hoc. ** *p* *<* *0.01,* *** *p* *<* *0.001*, as indicated. **Fig.** **S3.** ASK1-K716R protects tight junction proteins in endothelial cells 3 days following TBI. A Representative images of CD31^+^ (green) and Occludin^+^ (red) immunofluorescent staining. B Representative images of CD31^+^ (green) and VE-Cadherin^+^ (red) immunofluorescent staining. Scale bar, 20 μm. **Fig.** **S4.** ASK1-K716R does not alter the population of adaptive immune cell population in peripheral immune cells 3 days following TBI. A Gating strategy for immune cells in the peripheral blood and spleen. B Quantification of the numbers of immune cells in the blood. C Quantification of the numbers of immune cells in the spleen. *n* = 5 WT for sham, *n* = 6 for ASK1-K716R sham, *n* = 8 for WT TBI, *n* = 9 for ASK1-K716R TBI. All data from male mice are presented as the mean ± SD. one-way ANOVA test and Bonferroni post hoc. ns, no significance. **Fig.** **S5.** Effect of ASK1-K716R on early white matter injury at day 3 post-TBI. A A schematic diagram of the regions of interest in CTX, EC, and STR. B-C Representative images of MBP (green)/SMI32 (red) (B), and NF200 (purple) immunofluorescent staining (C). Scale bar, 100 μm. D-F Quantification of the fluorescence intensity of MBP (D), SMI32/MBP (E), and NF200 (F) in the CTX, EC, and STR. *n* = 4 for WT sham, *n* = 4 for ASK1-K716R sham, *n* = 7 for WT TBI, *n* = 7 for ASK1-K716R TBI). All data from male mice are presented as the mean ± SD. one-way ANOVA test and Bonferroni post hoc. * *p* *<* *0.05,* ** *p* *<* *0.01,* *** *p* *<* *0.001,* ns, no significance, as indicated. **Fig. S6.** Spatial learning and memory assessment 30 to 34 days after TBI using the Morris water maze. A Representative traces in the learning phase (upper panel) and memory test (lower panel). B Escape latency to find the hidden platform in the learning phase. *n* = 8 for WT sham, *n* = 8 for ASK1-K716R sham, *n* = 12 for WT TBI, *n* = 12 for ASK1-K716R TBI. All data from male mice are presented as the mean ± SD. Two-way ANOVA repeated measurement. * *p*

## Data Availability

The datasets generated during and/or analyzed during the current study are available from the corresponding author on reasonable request.

## Abbreviations

TBI: Traumatic brain injury
BBB: Blood–brain barrier
DAMP: Damage-associated molecular pattern
ASK1: Apoptosis signal-regulating kinase 1
MAPK: Mitogen-activated protein kinase
JNK: C-Jun N-terminal kinase
KD: Kinase domain
K716R: Map3k5-e site-specific mutation
WT: Wild-type
CCI: Controlled cortical impact
PVDF: Polyvinylidene difluoride
TUNEL: Terminal deoxynucleotidyl transferase dUTP nick end labelling
3D: 3-Dimensional
EB: Evans blue
TCA: Trichloroacetic acid
qPCR: Quantitative real-time PCR
CAPs: Compound action potentials
HBSS: Hank’s balanced salt solution
PBS: Phosphate-buffered saline
TCSSNT: Three‑chamber sociability and social novelty test
MWM: Morris water maze test
ANOVA: Analysis of variance
VE-cadherin: Vascular endothelial cadherin
WMI: White matter injury
EC: External capsule
NOR: Nodes of Ranvier
Caspr: Contactin-associated protein
CC: Corpus callosum
TAK1: Transforming growth factor-β-activated protein kinase 1
TAB1: TAK1 binding protein 1
MLK: Mixed lineage kinase
