# Supplementary material for: ASK1-K716R reduces neuroinflammation and white matter injury via preserving blood–brain barrier integrity after traumatic brain injury
Source: J Neuroinflammation. 2023 Oct 24;20:244. doi: 10.1186/s12974-023-02923-6 (PMC10594934; doi:10.1186/s12974-023-02923-6)
Supplement: Supplementary file 1 — Additional file 1: Table S1. Primer sequences used in real-time PCR analysis. Fig. S1. Partial DNA sequencing after ASK1-K716R and p-ASK1 expressed in brain cells. A A schematic representation of the nucleotide substitution (-AAGGAAATC- to -AGAGAAATA-) at site 716, resulting in the replacement of lysine with arginine during translation. B Representative immunofluorescence images displaying the colocalization of microglia (Iba1+), astrocytes (GFAP+), and neurons (NeuN+) with p-ASK1 in cortex of both WT TBI and ASK1-K716R TBI mice. Scale bar, 50 μm. Fig. S2. Effect of ASK1-K716R on neuronal apoptosis at day 3 post-TBI. A A schematic diagram showing the regions of interest around the lesioned site. Scale bar, 50 μm. The rectangle illustrates where images were taken. B Representative images of TUNEL staining in the cortical region around the lesioned site. C Quantification of the number of TUNEL+ neurons per hemisphere (n = 5 WT sham, n = 6 ASK1-K716R sham, n = 6 WT TBI, n = 6 ASK1-K716R TBI). All data from male mice are presented as the mean ± SD. One-way ANOVA test and Bonferroni post hoc. ** p < 0.01, *** p < 0.001, as indicated. Fig. S3. ASK1-K716R protects tight junction proteins in endothelial cells 3 days following TBI. A Representative images of CD31+ (green) and Occludin+ (red) immunofluorescent staining. B Representative images of CD31+ (green) and VE-Cadherin+ (red) immunofluorescent staining. Scale bar, 20 μm. Fig. S4. ASK1-K716R does not alter the population of adaptive immune cell population in peripheral immune cells 3 days following TBI. A Gating strategy for immune cells in the peripheral blood and spleen. B Quantification of the numbers of immune cells in the blood. C Quantification of the numbers of immune cells in the spleen. n = 5 WT for sham, n = 6 for ASK1-K716R sham, n = 8 for WT TBI, n = 9 for ASK1-K716R TBI. All data from male mice are presented as the mean ± SD. one-way ANOVA test and Bonferroni post hoc. ns, no significance. Fig. S5. Effect o [file 12974_2023_2923_MOESM1_ESM.docx]

**ASK1-K716R reduces neuroinflammation and white matter injury via preserving blood-brain barrier integrity after traumatic brain injury**

Shan Meng^1^, Hui Cao^1^, Yichen Huang^1^, Ziyu Shi^1^, Jiaying Li^1^, Yana Wang^1^, Yue Zhang^1^, Suning Chen^1^, Hong Shi^2*^, and Yanqin Gao^1*^

^1^State Key Laboratory of Medical Neurobiology, MOE Frontiers Center for Brain Science, and Institutes of Brain Science, Fudan University, Shanghai 200032, China

^2^Department of Anesthesiology, Shanghai Pulmonary Hospital, School of Medicine, Tongji University, Shanghai 200433, China

Address Correspondence to

Dr. Yanqin Gao,

State Key Laboratory of Medical Neurobiology, Fudan University, Shanghai 200032. China,

E-mail address: [yqgao@shmu.edu.cn](mailto:yqgao@shmu.edu.cn)

Or

Dr. Hong Shi,

Department of Anesthesiology, Shanghai Pulmonary Hospital, School of Medicine, Tongji University, Shanghai 200433, China,

Email: [ada-shi@139.com](mailto:ada-shi@139.com)

**Supplementary materials**

**Table S1** Primer sequences used in real-time PCR analysis

| Primer |  | Sequence |
| --- | --- | --- |
| *Map3k5* | F  R | 5'-CGTGCTGGACCGTTTTTACAA-3'  5'-TGTCACCATGTAGGGGATGAAG-3' |
| *IL-1b* | F  R | 5'-CTCCATGAGCTTTGTACAAGG-3'  5'-TGCTGATGTACCAGTTGGGG-3' |
| *TNF-a* | F  R | 5'-GACCCTCACACTCAGATCATCTTCT-3'  5'-CCTCCACTTGGTGGTTTGCT-3' |
| *CD16* | F  R | 5'-ACACATGTTCTCTGGGAAATC-3'  5'-AGTGCATCATCGTTGTTCATA-3' |
| *CD11b* | F  R | 5'-CCAAGACGATCTCAGCATCA-3'  5'-TTCTGGCTTGCTGAATCCTT-3' |
| *CCL-3* | F  R | 5'- TGTACCATGACATCTGCAAC -3'  5'- CAACGATGAATTGGCGTGGAA -3' |
| *iNOS* | F  R | 5'-CAAGCACCTTGGAAGAGGAG-3'  5'-CCTTTCAGTCCTTTGCAAGC-3' |
| *TGF-b* | F  R | 5'-TGCGCTTGCAGAGATTAAAA-3'  5'-CGTCAAAAGACAGCCACTCA-3' |
| *Ym1/2* | F  R | 5'-CAGGGTAATGAGTGGGTTGG-3'  5'-CAGGGTAATGAGTGGGTTGG-3' |
| *GAPDH* | F  R | 5'-CTGCCCAGAACATCATCCCT-3'  5'-TGAAGTCGCAGGAGACAACC-3' |

**Supplementary Figures:**


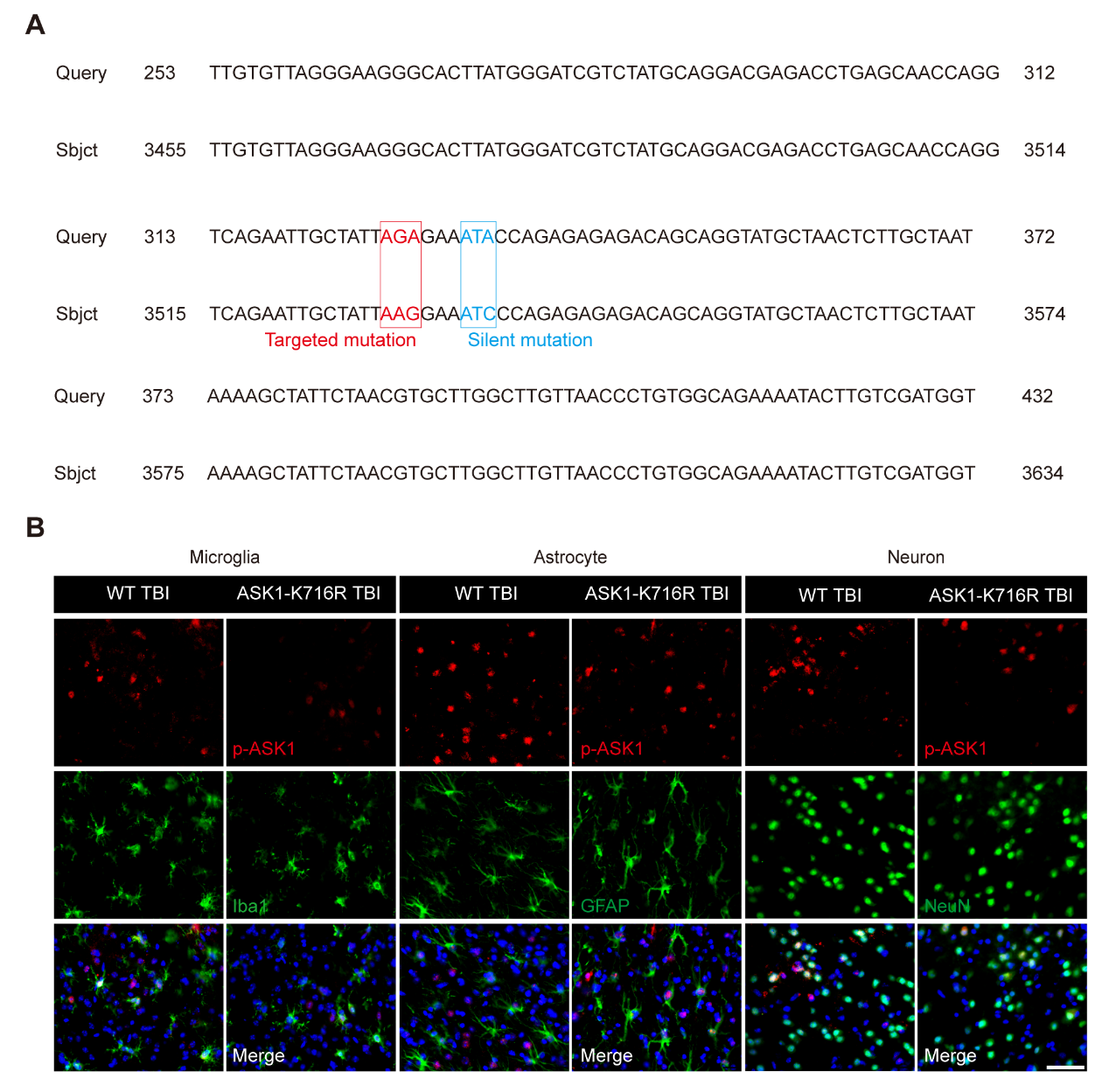


**Fig. S1 Partial DNA sequencing after ASK1-K716R and p-ASK1 expressed in brain cells.** **A** A schematic representation of the nucleotide substitution (-AAGGAAATC- to -AGAGAAATA-) at site 716, resulting in the replacement of lysine with arginine during translation. **B** Representative immunofluorescence images displaying the colocalization of microglia (Iba1^+^), astrocytes (GFAP^+^), and neurons (NeuN^+^) with p-ASK1 in cortex of both WT TBI and ASK1-K716R TBI mice. Scale bar, 50 μm.


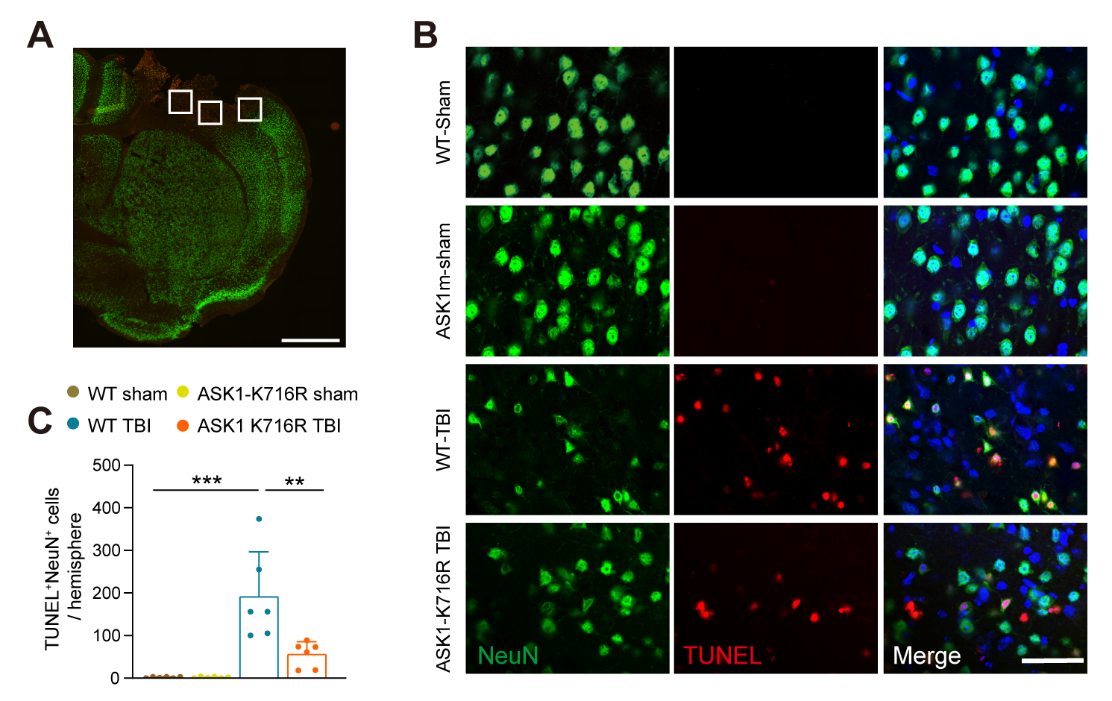


**Fig. S2** **Effect of ASK1-K716R on neuronal apoptosis at day 3 post-TBI.** **A** A schematic diagram showing the regions of interest around the lesioned site. Scale bar, 50 μm. The rectangle illustrates where images were taken. **B** Representative images of TUNEL staining in the cortical region around the lesioned site. **C** Quantification of the number of TUNEL^+^ neurons per hemisphere (n = 5 WT sham, n = 6 ASK1-K716R sham, n = 6 WT TBI, n = 6 ASK1-K716R TBI). All data from male mice are presented as the mean ± SD. One-way ANOVA test and Bonferroni post hoc. *** p < 0.01, *** p < 0.001*, as indicated.


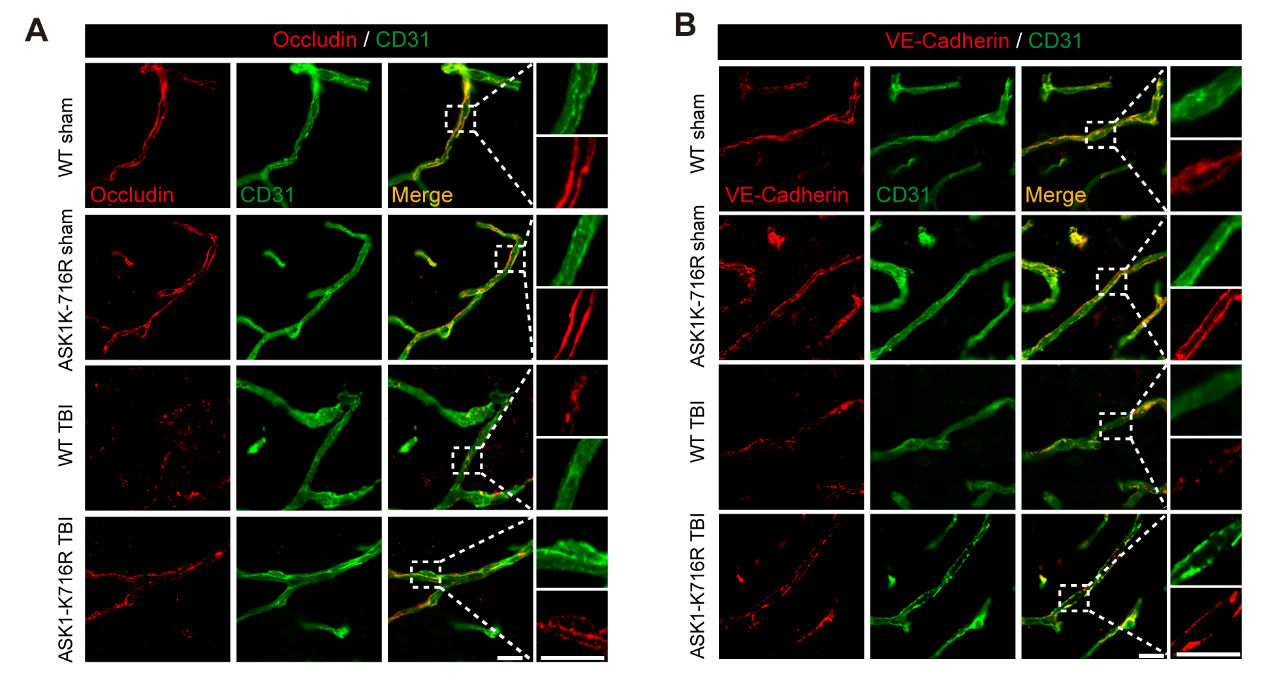


**Fig. S3** **ASK1-K716R protects tight junction proteins in endothelial cells 3 days following TBI. A** Representative images of CD31^+^ (green) and Occludin^+^ (red) immunofluorescent staining. **B** Representative images of CD31^+^ (green) and VE-Cadherin^+^ (red) immunofluorescent staining. Scale bar, 20 μm.


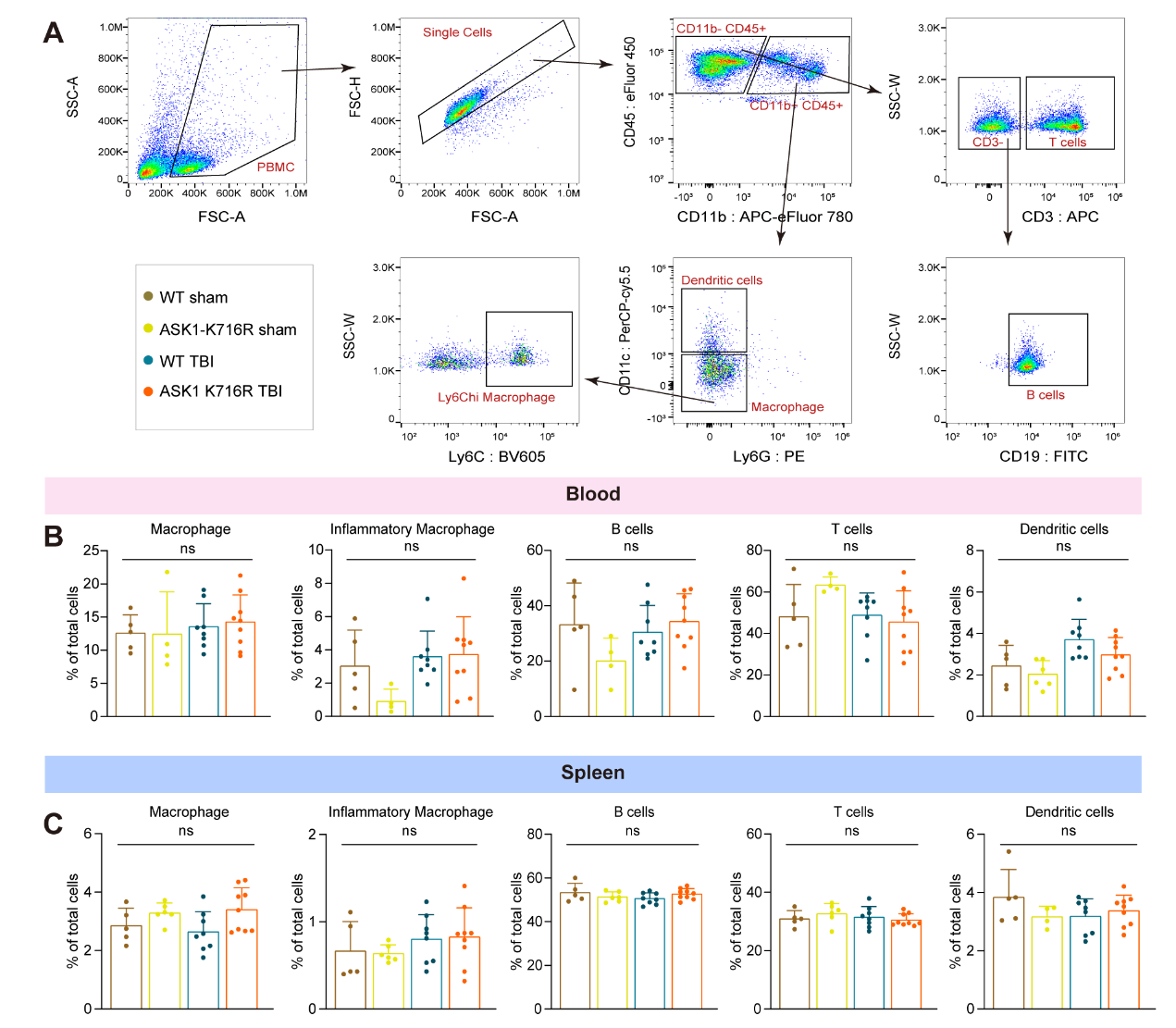


**Fig. S4** **ASK1-K716R does not alter the population of adaptive immune cell population in peripheral immune cells 3 days following TBI.** **A** Gating strategy for immune cells in the peripheral blood and spleen. **B** Quantification of the numbers of immune cells in the blood. **C** Quantification of the numbers of immune cells in the spleen. n = 5 WT for sham, n = 6 for ASK1-K716R sham, n = 8 for WT TBI, n = 9 for ASK1-K716R TBI. All data from male mice are presented as the mean ± SD. one-way ANOVA test and Bonferroni post hoc. ns, no significance.


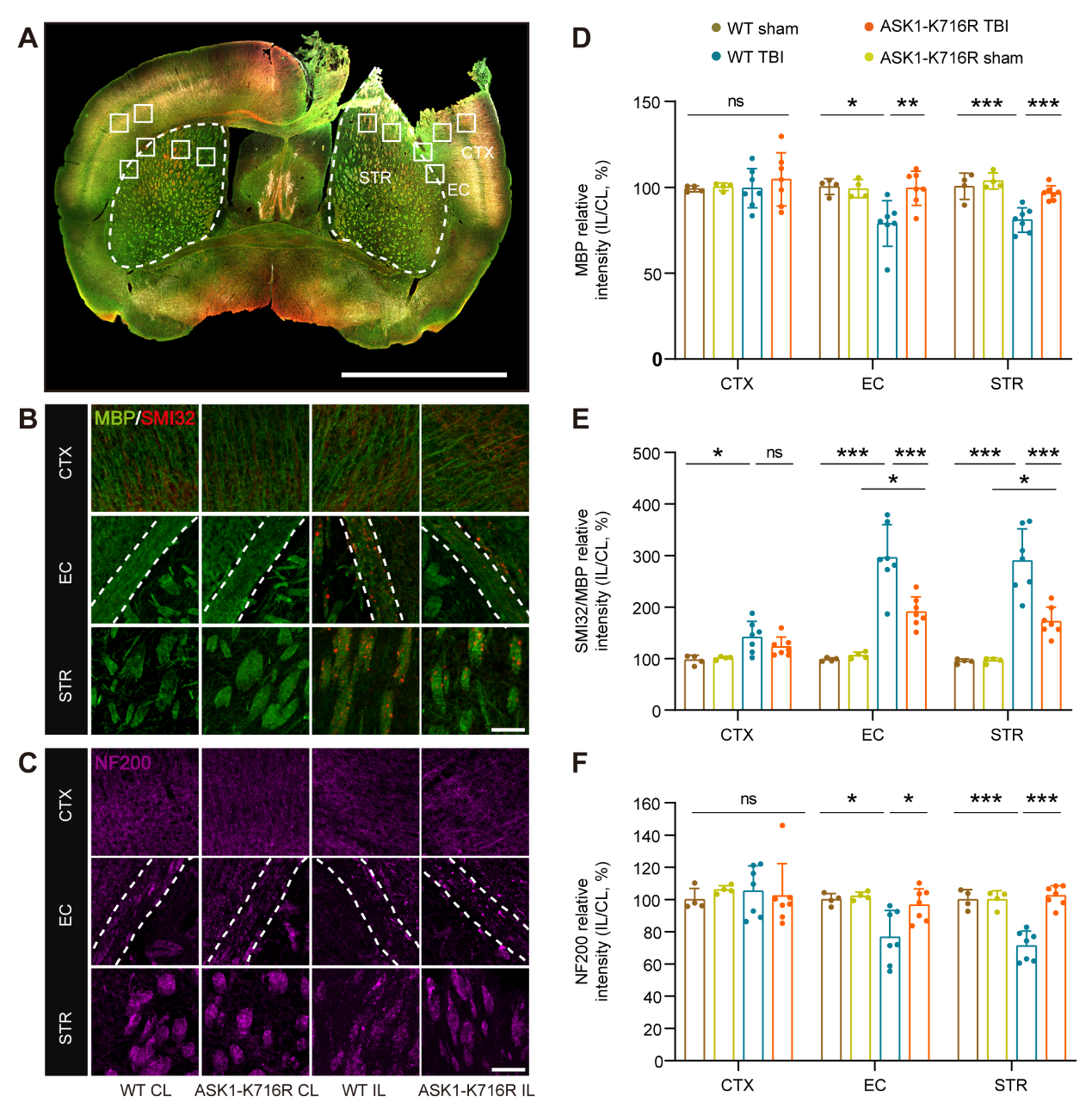


**Fig. S5 Effect of ASK1-K716R on early white matter injury at day 3 post-TBI.** **A** A schematic diagram of the regions of interest in CTX, EC, and STR. **B-C** Representative images of MBP (green)/SMI32 (red) **(B)**, and NF200 (purple) immunofluorescent staining **(C)**. Scale bar, 100 μm. **D-F** Quantification of the fluorescence intensity of MBP (D), SMI32/MBP (E), and NF200 (F) in the CTX, EC, and STR. n = 4 for WT sham, n = 4 for ASK1-K716R sham, n = 7 for WT TBI, n = 7 for ASK1-K716R TBI). All data from male mice are presented as the mean ± SD. one-way ANOVA test and Bonferroni post hoc. ** p < 0.05, ** p < 0.01, *** p < 0.001,* ns, no significance, as indicated.


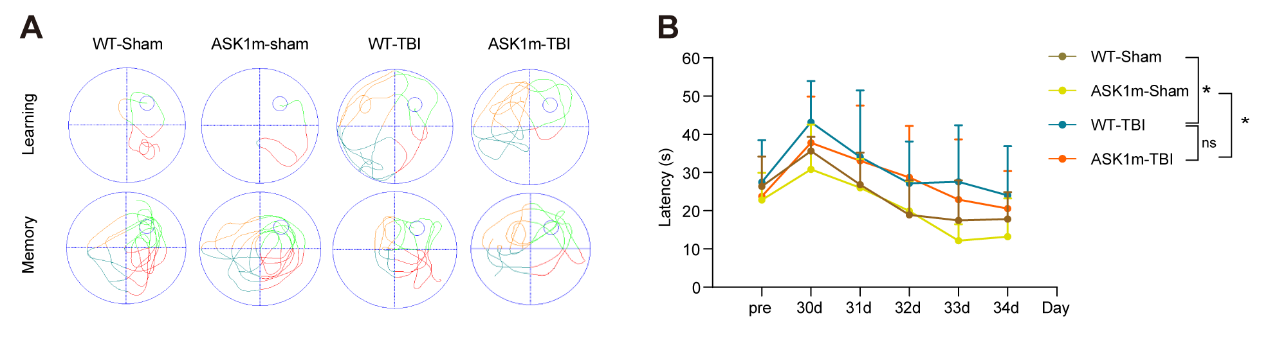


**Fig. S6 Spatial learning and memory assessment 29 to 34 days following TBI using the Morris water maze. A** Representative traces in the learning phase (upper panel) and memory test (lower panel). **B** Escape latency to find the hidden platform in the learning phase. n = 8 for WT sham, n = 8 for ASK1-K716R sham, n = 12 for WT TBI, n = 12 for ASK1-K716R TBI. All data from male mice are presented as the mean ± SD. Two-way ANOVA repeated measurement. ** p < 0.05,* ns, no significance, as indicated.
